# Walnut Protein Peptide Nanoparticles with Protective Mineralization: Resveratrol Encapsulation, Intestinal-Targeted Delivery and Synergistic Antioxidant Activity

**DOI:** 10.3390/foods14244310

**Published:** 2025-12-14

**Authors:** Jingwen Hou, Chao Liu, Chaoting Wen, Min Liu, Chunyan Xiang, Mengxue Fang, Liangxiao Zhang, Peiwu Li

**Affiliations:** 1 Xinjiang Key Laboratory of Agro-Products Quality & Safety, Xinjiang Academy of Agricultural Sciences, Urumqi 830091, China; 2 Key Laboratory of Biology and Genetic Improvement of Oil Crops, Ministry of Agriculture and Rural Affairs, Laboratory of Risk Assessment for Oilseed Products (Wuhan), Ministry of Agriculture and Rural Affairs, Quality Inspection and Test Center for Oilseed Products, Ministry of Agriculture and Rural Affairs, Key Laboratory of Edible Oil Quality and Safety, State Administration for Market Regulation, Oil Crops Research Institute, Chinese Academy of Agricultural Sciences, Wuhan 430062, China; 3 College of Food Science and Technology, Huazhong Agricultural University, Wuhan 430070, China; 4 College of Food Science and Engineering, Yangzhou University, Yangzhou 225127, China; 5 Wuhan Institute for Food and Cosmetic Control, Wuhan 430040, China; 6 Hubei Hongshan Laboratory, Wuhan 430070, China; 7 College of Food Science and Engineering, Nanjing University of Finance and Economics/Collaborative Innovation Center for Modern Grain Circulation and Safety, Nanjing 210023, China; 8 Zhongyuan Research Center, Chinese Academy of Agricultural Sciences, Xinxiang 453500, China; 9 Xianghu Laboratory, Hangzhou 311231, China

**Keywords:** walnut protein peptide, resveratrol, biomimetic mineralization, intestinal-target delivery, antioxidant activity

## Abstract

Resveratrol (RES) suffers from low bioavailability and poor gastrointestinal stability, limiting its health benefits. To overcome these challenges, we developed biomimetic mineralized nanoparticles based on walnut protein peptides (WPP-RES@CaP) for intestinal-targeted RES delivery. WPP with a 31.83% degree of hydrolysis was optimal for RES encapsulation. Subsequent mineralization with 5 mM Ca^2+^ significantly enhanced the encapsulation efficiency (EE) to 95.86%, compared to 73.69% for non-mineralized WPP-RES nanoparticles. The particle size and zeta potential of WPP-RES@CaP were 795 ± 16 nm and −27 ± 1 mV, respectively. Beyond the initial hydrophobic and π-π interactions, mineralization introduced additional stabilizing forces, including metal–ligand coordination, salt bridges, and electrostatic interactions, which collectively enhanced the structural integrity and RES retention of WPP-RES@CaP. During in vitro gastrointestinal digestion, the formation of a CaP shell protected RES and WPP from excessive degradation in the gastric phase. The 77.57% RES in WPP-RES@CaP was continuously released in the intestinal phase, which was higher than that of WPP-RES (49.73%). Meanwhile, the introduction of Ca^2+^ promoted the antioxidant activity of WPP-RES@CaP, which demonstrated higher DPPH and ABTS radical-scavenging activity assays than WPP-RES both before and after digestion. It was probably due to the synergistic effect of more released RES, antioxidant-free amino acids, and peptides. This mineralized peptide-based system provided a strategy for improving the delivery of hydrophobic bioactive compounds in functional foods.

## 1. Introduction

Resveratrol (3,5,4′-trihydroxy-trans-stilbene, RES) is a natural polyphenolic compound renowned for its potent antioxidant, anti-inflammatory, anticancer, and cardioprotective properties [1]. However, its practical applications are significantly hindered by inherent physicochemical limitations, including low water solubility, poor photochemical stability, and limited bioavailability [2]. Following oral administration, a substantial portion of RES undergoes degradation in the acidic gastric environment or is prematurely metabolized before reaching the primary absorption site (small intestine), thereby compromising its physiological efficacy, particularly its antioxidant activity [3].

Nanodelivery systems have emerged as a promising approach to overcoming these challenges [4,5]. Among various carriers, food-derived protein peptides are especially attractive due to their biocompatibility and intrinsic capacity to self-assemble into nanoscale structures. For instance, we previously developed RES-loaded nanogels using transglutaminase-crosslinked soybean peptides, which enhanced the in vitro intestinal release profile [6]. Similarly, Wang et al. [7] utilized peanut protein peptides and glucan conjugates to construct RES emulsions, improving its bioavailability after digestion. Walnut protein peptides (WPPs) were generated through enzymatic hydrolysis of walnut proteins, exhibiting amphiphilic characteristics and a strong self-assembly ability, making it an ideal candidate for encapsulating hydrophobic bioactive compounds such as RES. Asadi et al. [8] prepared curcumin walnut protein nanoparticles encapsulated by electrospray technology and mentioned that the antioxidant activity after in vitro digestion was significantly higher than that before digestion. Zhao et al. [9] also fabricated a walnut protein hydrolysate-Fe-vitamin C complex, proving its enhanced iron bioavailability in CaCO-2 cells. Furthermore, WPPs possess distinct physiological functions, including antiaging [10], antioxidant [11], and antifatigue effects [12]. Thus, WPPs not only serve as an effective delivery vehicle but also contribute synergistically to the overall bioactive capacity of the system [13].

However, a critical limitation of peptide-based carriers is their vulnerability to the harsh gastrointestinal environment. The acidic pH and proteolytic enzymes (e.g., pepsin) in the stomach can denature peptides and disrupt the nanostructure, resulting in the premature release and degradation of the payload [14]. To fortify the WPP-RES complex, we deposited a protective calcium phosphate (CaP) shell onto its core using a biomimetic mineralization strategy [15,16]. Such mineralized core–shell structures have shown great promise in drug delivery due to their pH-responsive dissolution behavior [17]. Firstly, during the gastric digestion process, CaP forms a dense physical barrier on the outer layer of the nanoparticles, which can prevent H^+^ from penetrating in the acidic gastric environment, avoid the excessive denaturation of peptides, and also reduce the direct contact between RES and H^+^ for degradation [18]. Secondly, the steric hindrance effect and charge characteristics of the CaP shell can repel pepsin, significantly reducing the large-scale enzymatic hydrolysis of the core peptide chain during gastric digestion [19]. This ensures that the delivery of RES nanoparticles is relatively intact and prevents the large-scale release of RES during gastric digestion. During intestinal digestion, CaP gradually dissociates in a neutral-to-alkaline intestinal environment, which facilitates the precise release of RES at the intestinal absorption site, providing dual protection and targeted release guarantee for the effective delivery of RES and antioxidant peptides [17].

This study aimed to engineer a robust, intestinal-targeted delivery system for RES by constructing biomimetic mineralized nanoparticles based on WPPs. We systematically investigated the formation, physicochemical properties, and structural evolution of the WPP-RES@CaP nanoparticles. Their performance was rigorously evaluated, focusing on the encapsulation efficiency, in vitro controlled release profile under simulated gastrointestinal conditions, and the preservation/synergistic enhancement of antioxidant activity. We demonstrate that this mineralized peptide-based platform effectively addresses the key challenges of RES delivery, offering a promising strategy for enhancing the bioavailability and efficacy of hydrophobic bioactive compounds in functional foods.

## 2. Materials and Methods

### 2.1. Materials

RES with a purity of ≥95% was obtained from Shanghai Yuanye Biotechnology Co., Ltd. (Shanghai, China). Walnut protein with a purity of ≥80% was obtained from Shaanxi Taizhikang Biotechnology Co., Ltd. in Xi’an, China. Alkaline protease (activity: 400,000 U/g) was purchased from Angel Enzyme Co., Ltd. in Yichang, China. The suppliers of pancreatic enzymes from the porcine pancreas (200 U/mL) and pepsin from porcine gastric mucosal pepsin (4000 U/mL) were purchased from Suzhou Xiaodong Yijian Instrument Co., Ltd. in Suzhou, China. All the reagents used in the experiment were analytical chemical reagents.

### 2.2. Preparation of WPPs

The walnut proteins were dissolved in deionized water to prepare a 5% (*w*/*v*) walnut protein solution. After adjusting the pH of the walnut protein solution to 9.0 with 10% (*w*/*v*) NaOH and saturated NaHCO_3_, it was kept in a water bath at 55 °C for 15 min. Then, alkaline protease was added at an enzyme-to-walnut protein mass ratio of 1:10 (*w*/*w*) to hydrolyze the proteins, followed by thorough stirring to homogenize the mixture. After hydrolysis at 55 °C for 1 to 6 h, samples were taken once every hour and placed in a boiling water bath for 15 min to terminate the enzymatic hydrolysis reaction. After cooling, samples were centrifuged at 8000 rpm for 15 min to obtain WPPs, and a portion of the WPP supernatant was freeze-dried for further experiments. According to the method of Nielsen et al. [17], the degree of hydrolysis (DH) of WPPs was quantitatively analyzed. Briefly, a solution was prepared by dissolving 7.62 g of borax and 200 mg of sodium dodecyl sulfate (SDS) in 150 mL of deionized water. After OPA was completely dissolved, the solution was transferred to the aforementioned aqueous solution. Subsequently, 176 mg of 1, 4-dimercaptosuccinol was added to the freshly prepared solution. Finally, deionized water was added until the solution reached 200 mL. A standard serine solution at a concentration of 0.9516 mM was prepared by dissolving 10 mg of serine in 100 mL of deionized water. In the sample group, 400 μL of WPP solution was added to a test tube containing 3 mL of OPA reagent. This mixture was thoroughly rotated and allowed to stand for 2 min. For the standard group, the WPP solution was replaced with serine standard solution, and for the blank group, the WPP solution was replaced with deionized water. The absorbance at 340 nm was immediately read. The DH value was determined by Equation (1).

(1) $$\mathrm{DH}(\%)=\frac{h}{\mathrm{hot}}=\frac{(A\;-\;B)/(C\;-\;B)\times 0.9516\;-\;\beta }{\alpha \;\times \;\mathrm{hot}}\;$$

where A is the absorbance of the sample well, B is the absorbance of the blank well, C is the absorbance of the serine standard well, h is the content of free peptide bonds after hydrolysis, hot is the total content of peptide bonds in the protein sample, SPI is 7.8 mmoL/L, α and β are correction factors, and the α and β of SPI are 0.97 and 0.342, respectively.

### 2.3. Preparation of WPP-RES

Firstly, RES was dissolved in absolute ethanol to prepare the RES solution at a concentration of 30 mg/mL. Then, 1 mL of RES solution was slowly dropped into 50 mL of the WPP solution and sonicated at 50 °C for 30 min. Then, the mixture solution was transferred to a constant temperature water bath at 50 °C and stirred for 2 h to promote the complete interaction between RES and WPP. Subsequently, the solution was placed in a vacuum oven at 50 °C for 30 min to remove anhydrous ethanol, and finally WPP-RES nanoparticles were obtained.

### 2.4. Encapsulation Efficiency of RES

The EE of RES was indirectly determined by measuring the contents of unencapsulated RES in the WPP-RES suspension [18]. To extract unencapsulated RES, 1 mL of the WPP-RES sample was mixed with 4 mL of methanol and centrifuged at 10,000 rpm for 10 min. The resulting supernatant was passed through a 0.22 μm filter. The filtered solution was concentrated to 1 mL under a nitrogen stream and placed in an injection vial. Analysis was performed on an Agilent 1200 HPLC system with a C18 column (5 μm, 260 mm × 4.6 mm) under the following conditions: UV detection at 306 nm, a mobile phase of acetonitrile–acetic acid water (25:5, *v*/*v*), a column temperature of 25 °C, and a flow rate of 1.0 mL/min.

(2) $$\mathrm{EE}\;(\%)=\frac{m_{t}-m_{ue}}{m_{t}}\;\times \;100\%$$

where m_t_ is the total amount of RES added, and m_ue_ is the quantity of unencapsulated RES.

### 2.5. Preparation of WPP-RES@CaP

WPP-RES prepared from WPP hydrolyzed for 5 h exhibited the highest EE. On this basis, a series of calcium precursor systems were prepared by dissolving CaCl_2_ of different concentrations (1, 3, 5, 7, and 9 mM) in it. Under magnetic stirring at room temperature, Na_2_HPO_4_ was dropped into the calcium precursor system at a Ca/P molar ratio of 1.67 [20]. The pH value of the reaction system was maintained at 7.5 throughout the entire process. After Na_2_HPO_4_ was completely added, the mixed systems were stirred in the dark for 1 h to ensure complete mineralization The resulting milky suspension was then centrifuged (12,000 rpm, 10 min, 4 °C) to collect the mineralized WPP-RES@CaP nanoparticles. Then the encapsulation rate EE of WPP-RES@CaP was determined by method 2.4.

### 2.6. Particle Size, Polydispersity Index, and Zeta Potential

According to the method of Tao et al. [21], WPP, WPP-RES, and WPP-RES@CaP were diluted 100 times and then the mean particle size, polydispersity index (PDI), and zeta potential were determined using a dynamic light scattering (DLS) analyzer (Malvern Pro, instruments Inc., Malvern, UK).

### 2.7. Scanning Electron Microscope (SEM)

In order to observe the surface structures of the WPP, WPP-RES, and WPP-RES@CaP, the nanoparticles were first freeze-dried and then fixed on aluminum stakes after gold spraying. SEM images were observed with a SEM instrument (Zeiss GeminiSEM 360, Oberkochen, Germany).

### 2.8. Transmission Electron Microscope (TEM)

In order to observe the shape of WPP, WPP-RES, and WPP-RES@CaP, 0.5 μL of the nanoparticles were dropped onto a carbon-coated copper mesh and completely dried at 37 °C. Subsequently, TEM images were observed with a TEM instrument (JEM-F200, JEOL, Tokyo, Japan) at an accelerated voltage of 80 kV.

### 2.9. Surface Hydrophobicity

Surface hydrophobicity (H_0_) was determined according to the method described by Liu et al. [22]. WPP, WPP-RES, and WPP-RES@CaP nanoparticles were first dissolved in 0.01 mol/L H_3_PO_4_ buffer (pH 7.0) to prepare serial concentrations ranging from 0 to 10 mg/mL. A 20 μL aliquot of 8 mmol/L 8-anilino-1-naphthalenesulfonic acid (ANS) solution was then mixed with 2 mL of the nanoparticle solutions via vortexing, and the mixture was incubated at room temperature for 3 min in the dark. Subsequently, the mixture was transferred to a 96-well microplate, and fluorescence intensity was measured using a multi-mode microplate reader (Spectramax i3x, Molecular Devices, San Jose, CA, USA), with detection parameters set as follows: an excitation wavelength of 390 nm, an emission wavelength of 470 nm, and a slit width of 5 nm. Finally, H_0_ was identified by using the slope of the linear regression model of the relationship between fluorescence intensity and protein content.

### 2.10. Fourier Transform Infrared Spectroscopy (FT-IR)

Referring to the method of Ma et al. [23], the secondary structure was analyzed by the KBr plate method using the FT-IR spectrophotometer (Japan-Shimadzu-IR Tracer 100, Kyoto, Japan). Data were collected by scanning 32 times with a resolution of 4 cm^−1^ in the range of 400–4000 cm^−1^ for WPP, WPP-RES, and WPP-RES@CaP.

### 2.11. Circular Dichroism Chromatography

Circular dichroism (CD) spectroscopy (J-1500, JASCO, Tokyo, Japan) was used to obtain the circular dichroism spectra of WPP, WPP-RES, and WPP-RES@CaP [24]. The path length is between 190 and 260 nm, with a length of 1 mm. Then, the secondary structure is analyzed using the CDNN 2.1 software.

### 2.12. Fluorescence Spectroscopy Analysis

The fluorescence spectra of the samples were obtained by dissolving WPP, WPP-RES, and WPP-RES@CaP to 1 mg/mL using a fluorescence spectrophotometer (LS45, PerkinElmer, Waltham, MA, USA) for the detection of their tertiary structure. The emission spectra were recorded at the excitation wavelength of 280 nm within the range of 300 to 450 nm. The excitation slit width is set at 5 nm, and the emission slit width is set at 10 nm. The scanning speed is maintained at 240 nm/min, and the acceleration voltage is 400 V [25].

### 2.13. X-Ray Diffraction

The nanoparticles, CaP, Na_2_HPO_3_, CaCl_2_, and physical mixtures of WPP and RES were characterized using an X-ray diffractometer (XRD) (Smart Lab 9 kW, Rigaku, Tokyo, Japan). The voltage was set to 40 kV, the current of the electron tube was set to 30 mA, the 2θ was set from 3° to 30°, and the step was set to 0.05° [26].

### 2.14. In Vitro Digestion of WPP-RES and WPP-RES@CaP

The simulated gastric digestive fluid (SGF) and intestinal digestive fluid (SIF) were prepared according to the method of Gao et al. [27]. Among them, pepsin from porcine gastric mucosa and pancreatic enzymes from porcine pancreas were added to SGF and SIF at concentrations of 4000 U/mL and 200 U/mL, respectively. First, the pH values of 100 mL of WPP-RES and WPP-RES@CaP were adjusted to 2.0 with 0.1 M HCl. Then, the same volume of SGF was added and shaken in a constant temperature shaker at 37 °C for 2 h. Then it was taken out and heated at 100 °C for 10 min to inactivate the enzyme. After the gastric digestion was completed, the pH value of the mixed solution was adjusted to 7.0 with saturated NaHCO_3_. Then, an equal volume of SIF was added to the mixed solution, and it was shaken in a constant temperature shaker at 37 °C for 3 h. After the digestion was completed, it was heated at 100 °C for 10 min to inactivate the enzyme. The digested sample was centrifuged at 4500 rpm for 10 min to obtain the supernatant.

At designated time points during the gastric (10, 20, 30, 60, 90, and 120 min) and intestinal (10, 20, 30, 60, 90, 120, 150, and 180 min) phases, 5 mL aliquots were withdrawn to monitor and calculate the cumulative release of RES. After collecting the supernatant, the released amount of RES was measured by method 2.3, and the cumulative release rate of RES at each time point was calculated according to Equation (3).

Q = R_i_/R_t_ (3)

where is R_i_ represents the amount of RES released at each sampling time and R_t_ is the total amount of RES released in the medium.

### 2.15. Free Amino Acids

According to Wang et al. [28], the nanoparticle digestion sample (0.2 g) was dissolved in 50 mL of 0.067 M sodium citrate buffer (pH 2.2). Subsequently, 1 mL of the dissolved sample was filtered, acidified with 100 μL of 0.2 mol/L hydrochloric acid, and filtered again through a 0.22 μm membrane. The contents of free amino acids of the final filtrate were analyzed with an automatic amino acid analyzer (LA8080, Hitachi High-Tech Analytical Science Co., Ltd., Tokyo, Japan).

### 2.16. DPPH Free Radical-Scavenging Activity Assay

The DPPH assay was conducted according to previously published methods [29]. Initially, 2 mL of the 0.2 mM DPPH ethanol solution was added to 2 mL of nanoparticle solution and vortexed to mix well. The reaction was conducted in a dark environment for 30 min, and the absorbance of the reaction solution was measured at 517 nm. For the blank group, the sample solution was replaced with distilled water. For the control group, the DPPH solution was replaced with an equal volume of ethanol [30]. The DPPH scavenging activity assay was calculated according to Equation (4).

(4) $$DPPH\;free\;radical\;scavenging\;activity\;assay\left(\%\right)=1-\frac{A_{2}-A_{1}}{A_{0}}\times 100\%$$

where A_0_, A_1_, and A_2_, respectively, represent the absorbance of the control group, the blank group, and the sample group.

### 2.17. ABTS Free Radical-Scavenging Activity Assay

The ABTS assay was conducted based on the method of Qi et al. [31]. Firstly, 5 mL of ABTS radical solution was prepared in the concentrations of 7 mM for the ABTS radical solution and 2.45 mM for the potassium persulfate and stored in the dark for 12 h for later use. Then, the ABTS free radical solution was diluted with deionized water to ensure its absorbance at 734 nm around 0.70 ± 0.02. Then, 150 μL of nanoparticles and 150 μL of ABTS free radical solution were, respectively, added to 96-well plates, mixed evenly, and reacted in the dark for 10 min. The absorbance was measured at 734 nm. For the blank group, the sample solution was replaced with distilled water. For the control group, the ABTS solution was replaced with an equal volume of water. The ABTS free radical-scavenging activity assay was calculated according to Equation (5).

(5) $$ABTS\;free\;radical\;scavenging\;activity\;assay\left(\%\right)=1-\frac{B_{2}-B_{1}}{B_{0}}\times 100\%$$

where B_0_, B_1_, and B_2_, respectively, represent the absorbance of the control group, the blank group, and the sample group.

### 2.18. Peptides Analysis

According to Fang et al.’s [32] previous study, The WPPs underwent desalting on a C18 column before being analyzed via peptide mass spectrometry utilizing an Ultimate 3000 RSL nano (USA). The mass spectrometer operated in DDA top 20 mode with a full scan *m*/*z* value range spanning from of 100 to 1500. The target value for AGC during full MS scans was set to 3 × 10^6^ charges. The maximum injection time allowed was set to 30 ms with a resolution of 60,000 at *m*/*z* 200. A precursor ion selection window of 1.4 *m*/*z* was maintained throughout fragmentation, achieved through high-energy collision dissociation (HCD), employing normalized collision energy settings of 28. Fragment ion scanning records exhibited resolutions of 15,000 with AGC values fixed at 1 × 10^5^ and maximum filling times capped at 50 ms. Dynamic exclusion functionality was enabled and configured for durations not exceeding 30 s. The UniProt Glycine Max database (http://www.UniProt.org/) was used to search the data. The activity score of antioxidant peptides was predicted via PeptideRanker (http://distilldeep.ucd.ie/PeptideRanker/), and their free radical-scavenging activity was predicted by AnOxPePred 1.0 (https://services.healthtech.dtu.dk/services) [33,34]. The uniform resource locator (URL) was accessed on 22 September 2025.

### 2.19. Molecular Docking

The three-dimensional structure of peptides was constructed using ChemBioDraw Ultra version 22.0 (PerkinElmer, USA). Molecular docking studies were conducted using Autodock Vina 1.5.6 docking software (La Jolla, CA, USA). The DPPH and ABTS structures were obtained from the PubChem database (https://pubchem.ncbi.nlm.nih.gov/). The ideal docking conformation is determined by choosing the conformation with the lowest binding energy. The interaction forces between receptor proteins and polypeptides were analyzed using Discovery Studio 4.5 Client (Dassault Systemes Biovia, San Diego, CA, USA), and the optimized interaction points were visualized in 2D graphs.

### 2.20. Statistical Analysis

The above experimental results were repeated three times, analyzed using IBM SPSS Statistics 26 (Chicago, IL, USA), and expressed as mean ± standard deviation. Origin 2024 (Northampton, MA, USA) is used for mapping.

## 3. Results and Discussion

### 3.1. The Encapsulation Efficiency of RES

**Figure 1:**
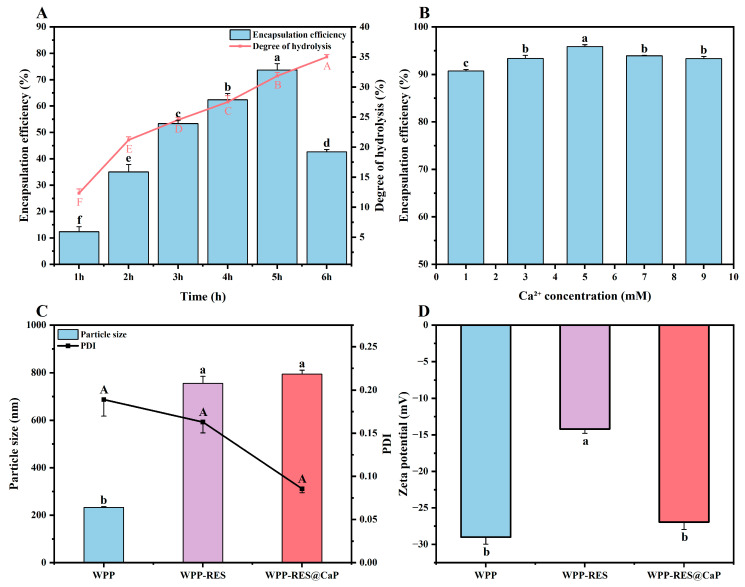
Encapsulation efficiency of RES and degree of hydrolysis of WPPs under different hydrolysis times (**A**). Encapsulation efficiency of RES under different Ca^2+^ concentrations (**B**). Particle size and PDI of WPPs, WPP-Res, and WPP-Res@CaP (**C**). Zeta potential of WPPs, WPP-RES, and WPP-RES@CaP (**D**). The different lowercase letters in (**A**,**B**) indicate significant differences in encapsulation efficiency between groups (*p* < 0.05). The different lowercase letters in (**C**,**D**) respectively indicate significant differences in particle size and zeta potential between groups (*p* < 0.05). The different uppercase letters in (**A**,**C**) respectively indicate significant differences in degree of hydrolysis and PDI between groups (*p* < 0.05).

Figure 1A showed the effect of hydrolysis time on the DH of WPPs and EE of RES. The DH showed a continuous upward trend with the extension of hydrolysis time. During the 6 h of the hydrolysis process, DH gradually increased from 12.33% to 35.00%. A longer hydrolysis time enables alkaline proteases to hydrolyze walnut proteins more thoroughly, exposing more internal protein structures and obtaining more free amino groups, thereby promoting a continuous increase in DH [35]. The EE showed a trend of first increasing and then decreasing with the change in hydrolysis time and the EE reached the peak of 73.69% at 5 h. However, when the hydrolysis time was prolonged to 6 h, the EE rapidly decreased to 42.64%. Enzymatic hydrolysis could affect the molecular weight of proteins. The higher the DH, the more subunit structures with lower molecular weights in walnut protein [36].

Between 1 and 2 h of hydrolysis, EE rapidly increased from approximately 10% to 35%. At this point, the alkaline protease only performed a preliminary cleavage on the walnut protein, and the large-molecule protein was degraded into a small amount of short peptides. The alkaline protease could specifically cleave the C-terminal peptide bond of hydrophobic amino acids, thereby exposing some hydrophobic groups (such as benzene rings and alkyl chains) in the peptide chain [26]. At the same time, some polar amino acids were retained to initially form an amphoteric peptide structure. These amphoteric peptides could rapidly self-assemble into nanoparticles through hydrophobic and other interactions and provide initial encapsulation sites for RES [37,38]. Therefore, a rapid improvement in EE was achieved. However, at this point, the degree of hydrolysis was limited, which might lead to an insufficient amount of amphoteric peptides formed, thus setting an upper limit on EE. As hydrolysis continued (2–5 h), the growth of EE entered a slow stage. At this point, a large number of macromolecular proteins might have already been enzymically hydrolyzed into peptides. However, the recognition and cleavage rate of short peptides by alkaline proteases was not as good as that of proteins. Therefore, the generation rate of amphiphilic peptides slowed down, which in turn led to a slower growth rate of EE.

The hydrolyzed amphiphilic WPPs could self-assemble into nanoparticles in aqueous solution. During the self-assembly process, these peptides spontaneously aggregated inward in the hydrophobic region and outward in the hydrophilic region, thereby forming hydrophobic nuclei [39]. As a hydrophobic active substance, RES tended to be located in non-polar nuclei to avoid contact with water and reduce the contact between non-polar groups and water driven by the hydrophobic effect [40]. Similarly, Wang et al. [7] showed that partially enzymatically hydrolyzed lactoferricin peptides could form self-assembled nano-micelles, and the nano-micelles were able to encapsulate curcumin with high loading efficiency through hydrophobic interaction. Meanwhile, adjacent peptide chains could form a network structure through disulfide bond cross-linking, and then the RES could be restricted by the network cross-linking structure, thereby significantly improving the encapsulation efficiency of RES. As Liu et al. [41] stated, moderately hydrolyzed soybean peptides could promote aggregation to form a denser and more uniform gel network structure through hydrophobic interactions, hydrogen bonds, and disulfide bonds, which was more conducive to the encapsulation of hydrophobic substances. In addition, hydrolysis could increase the flexibility of peptides and the exposure of hydrophobic groups. The enhanced amphiphilicity of WPPs provides more binding sites for embedding with RES [35]. Therefore, 5 h of hydrolysis time was considered to have an ideal peptide composition in this regard. However, a subsequent decrease in EE was observed at 6 h, indicating that excessive hydrolysis was detrimental to encapsulation function. Prolonged enzymatic treatment cleaved medium-sized functionally active peptides into very short oligopeptides and free amino acids [42]. Although these small molecules are highly soluble, they lack the necessary structural integrity and amphiphilicity. Their limited number of functional groups reduced their ability to form stable interactions with RES molecules, resulting in a decrease in EE.

Compared with the unmineralized WPP-RES, the overall EE of WPP-RES@CaP prepared after mineralization was significantly improved (Figure 1B). This was because of the formation of the CaP shell, which played a crucial role in the biomimetic mineralization process. Ca^2+^, as the core cation of the CaP shell, could form CaP crystal units with phosphate ions through ionic bonds, which drives mineralization to form a dense shell layer. It can also coordinate and cross-link with the polar groups of WPP to enhance the mechanical strength and structural integrity of the CaP shell [43]. The CaP shell acted as a physical barrier, reducing the ionization and leakage of RES and effectively locking them inside the nanoparticles. On the other hand, WPP-RES and CaP might stabilize the conformation of the peptide chain through the synergistic effects of various forces such as metal coordination and salt bridges, further strengthening the structure of nanoparticles, thereby jointly enhancing EE. The specific combination mechanism is discussed in depth in Section 3.9. Similarly, Jiang et al. [44] also increased the ordered structure of soy proteins by chelating sodium tripolyphosphate with calcium ions, which further stabilized the stability of soy protein emulsion. The concentration of Ca^2+^ significantly influenced the EE of WPP-RES@CaP. It was obvious that WPP-RES@CaP reached the optimal EE (95.86%) at the Ca^2+^ concentration of 5 mM. When the calcium ion concentration was less than 5 mM, the availability of ions was limited, which might lead to the formation of a thin or incomplete inorganic shell, and cannot provide sufficient protection for WPP-RES. Thus, it failed to reach the maximum value of EE. In addition, an excessively high concentration of Ca^2+^ could lead to ion supersaturation, causing CaP to precipitate spontaneously in the system and failing to further enhance the coating efficiency of WPP-RES.

### 3.2. Particle Size, PDI, and Zeta Potential

The particle size, PDI, and zeta potential of the nanoparticles are shown in Figure 1C,D. The particle size of WPP, WPP-RES, and WPP-RES@CaP were 233 ± 4 nm, 755 ± 30 nm, and 795 ± 16 nm, respectively. The PDI were 0.19 ± 0.02, 0.16 ± 0.01, and 0.09 ± 0.01, respectively. The zeta potentials were −28.99 ± 0.97 mV, −14.21 ± 0.57 mV, and −26.93 ± 1.02 mV, respectively. After loading RES, the average particle size of WPP-RES increased significantly, and this increase evidenced the formation of a larger composite structure after the successful encapsulation of RES. This was consistent with the research results of Jiang et al. [45], in which they loaded curcumin and anthocyanin with α-whey protein peptides, and the particle size of nano-micelles increased. The further increase in the particle size of WPP-RES@CaP was caused by the spontaneous adsorption of positively charged mineral ions to the surface of WPP-RES through electrostatic interaction to form nano-mineral shells [46]. The increase in particle size might also be related to phosphate. Ca^2+^ and phosphate first rapidly combined through ionic bonds to form the initial crystal nuclei of amorphous calcium and phosphorus. These crystal nuclei then continued to grow through the continuous adsorption system of free Ca^2+^ and PO_4_^3−^ and gradually formed larger and denser CaP shells, thereby leading to an increase in size [43]. Although there was no significant difference (*p* < 0.05) in the PDI between WPP-RES and WPP-RES@CaP, it still showed a downward trend after mineralization. A lower PDI was more conducive to the dispersion of nanoparticles, so the distribution of WPP-RES@CaP was more uniform [41].

The zeta potential reflected the charged characteristics of the particle surface. The higher its absolute value, the stronger the electrostatic repulsion, and the higher its stability [47]. It was precisely this electrostatic repulsion that, to a certain extent, prevented the large-scale aggregation of WPPs and maintained the particle size of WPPs at a relatively low level, which was in line with the result of the particle size. The absolute value of the zeta potential of WPP-RES decreased, which might be because the hydrophobic groups of RES partially mask the charged carboxyl groups on the surface of WPPs, and the negative charge contributed by the hydroxyl groups of RES itself was limited, resulting in a decrease in the negative charge density on the particle surface [48]. After mineralization, the significantly higher absolute zeta potential of WPP-RES@CaP (−26.93 mV) compared to WPP-RES (−14.21 mV) indicates superior colloidal stability, which minimizes particle aggregation and may contribute to a more uniform behavior during digestion. However, the primary mechanism for its enhanced gastric protection is attributed to the physical barrier posed by the CaP shell, which impedes the penetration of H^+^ and pepsin, rather than electrostatic repulsion.

### 3.3. Morphological Analysis

**Figure 2:**
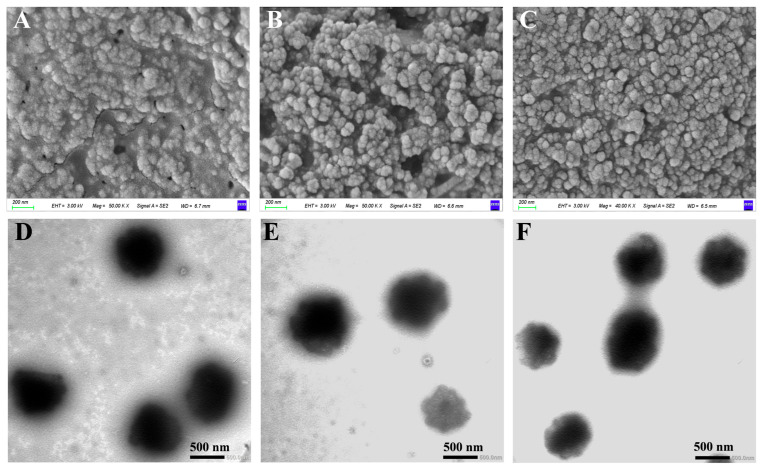
Morphological observation. Scanning electron microscope images of WPPs, WPP-Res, and WPP-Res@CaP, respectively (**A**–**C**). Transmission electron microscope images of WPPs, WPP-Res, and WPP-Res@CaP, respectively (**D**–**F**).

The morphology of WPPs, WPP-RES, and WPP-RES@CaP was observed by SEM (Figure 2A–C) and TEM (Figure 2D–F). In SEM images, WPPs presented an irregularly agglomerated particle morphology with a wide distribution, which was a typical manifestation of the spontaneous aggregation of protein peptide chains [49]. The nanoparticles of WPP-RES were closely interconnected and tended to aggregate, with a slightly rough surface texture. This change indicated that the loading of RES molecules promoted the aggregation and recombination of WPPs, thereby forming a more definite particle structure. WPP-RES@CaP was composed of spherical or nearly spherical nanoparticles with a denser surface, more uniform size distribution, and clearer boundaries between particles. The results clearly indicated that WPP determined this uniform distribution morphology through ligand-binding with calcium ions [50]. This phenomenon was similar to that observed by Fang et al. [51], in which the hydrolysis products of Manchu walnut powder and calcium ions also formed a dense aggregate structure.

The TEM images of WPPs revealed irregular and amorphous aggregates lacking distinct nanostructures. In contrast, more consolidated oval nanoparticles were observed in WPP-RES, which were characterized by a weakly stained hydrophilic outer layer. The emergence of these well-defined nanostructures from the amorphous WPP precursors suggested that the interaction between WPPs and RES facilitated the peptide self-assembly process, promoting the recombination of peptide chains to encapsulate hydrophobic RES molecules [52]. Notably, WPP-RES@CaP exhibited a distinct core–shell nanostructure, featuring a smooth and continuous outer layer enveloping a darker, uniformly thick ring surrounding the inner nanoparticle core. These observations indicated that the WPP-RES nanoparticles were uniformly and completely coated with an inorganic CaP mineral phase, signifying successful biomimetic mineralization [53,54]. The dense structure of WPP-RES@CaP not only enhanced the EE of RES but also provided an inorganic shell that acted as a protective barrier during gastrointestinal digestion. This mechanism prevented the excessive release of RES during gastric digestion while facilitating intestinal delivery for subsequent absorption.

### 3.4. Surface Hydrophobicity Analysis

**Figure 3:**
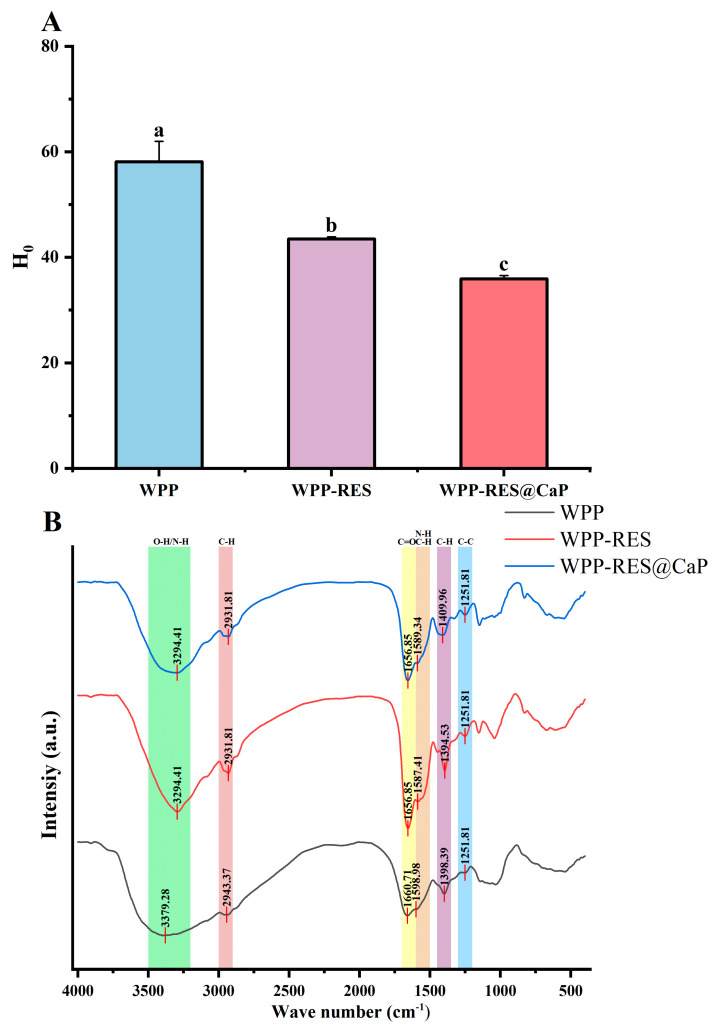
H_0_ values of WPPs, WPP-RES, and WPP-RES@CaP (**A**). FT-IR spectra of WPPs, WPP-RES, and WPP-RES@CaP (**B**). Different letters indicate significant differences (*p* < 0.05).

Surface hydrophobicity reflects the extent to which hydrophobic groups are exposed on protein surfaces, with higher H_0_ values indicating stronger hydrophobicity [55]. As illustrated in Figure 3A, the peptide chain structure of WPPs inherently contained hydrophobic residues, imparting a certain degree of surface hydrophobicity. Upon combination with hydrophobic RES, the H_0_ value for WPP-RES decreased to 43.49 compared with that of pure WPPs. The encapsulation of RES within WPPs through hydrophobic interactions led to the embedding of the hydrophobic aromatic rings of RES into the hydrophobic regions of WPPs. As a result, some originally exposed hydrophobic groups became internally buried, significantly reducing the surface hydrophobicity of WPP-RES relative to WPPs. Previous studies have reported that both covalent and non-covalent interactions between natural polyphenols and dietary proteins could reduce surface hydrophobicity due to phenol–protein interactions [56]. Similarly, Ji et al. [57] observed that the formation of fibrous structures in soy protein–curcumin complexes reduced surface hydrophobicity owing to the exposure of previously concealed regions. Furthermore, after biomimetic mineralization, the H_0_ value exhibited an additional decrease, which was likely due to the exposure of polar groups caused by electrostatic interactions or coordination between Ca^2+^ ions and non-polar residues within the WPP-RES complex [58]. Overall, this trend of reduced hydrophobicity is conducive to the encapsulation and stability of RES.

### 3.5. FT-IR Analysis

FT-IR spectroscopy was employed to analyze the intermolecular interactions and conformational changes in the proteins [59]. As shown in Figure 3B, the absorption peaks observed at 3500–3200 cm^−1^ (amide A band) indicated the presence of intermolecular hydrogen bonds, which primarily corresponded to O-H/N-H stretching vibrations [60]. The characteristic peak of WPPs appeared at 3379.28 cm^−1^, whereas those of WPP-RES and WPP-RES@CaP redshifted to 3294.41 cm^−1^ with increased intensity. This shift was attributed to hydrogen bond formation between RES and peptide chains in the WPPs, which reduced the O-H/N-H stretching vibration energy [32]. In the 2800–3000 cm^−1^ region (amide B band) representing C-H stretching vibrations [61], the characteristic peak of WPPs appeared at 2943.37 cm^−1^, while those of WPP-RES and WPP-RES@CaP were redshifted to 2931.81 cm^−1^. This shift likely resulted from hydrophobic interactions between WPPs and the aromatic rings of RES, which altered the electron cloud density around the C-H bond and reduced the vibration frequency [62]. WPP-RES@CaP exhibited stronger non-covalent interactions between WPPs and RES compared to WPP-RES.

The amide I band (1700–1600 cm^−1^), dominated by C=O stretching vibrations and associated with having a secondary structure, shifted from 1660.71 cm^−1^ to 1656.85 cm^−1^ after RES loading, indicating partial secondary structure rearrangement and enhanced C=O stretching intensity [63]. After loading RES, the peak position of the amide I band shifted from 1660.71 cm^−1^ to 1656.85 cm^−1^. Meanwhile, the strength of the amide I band is increased by stretching the C=O bond. The amide II band (1700–1600 cm^−1^) reflects the bending vibration of the N-H group and the stretching vibration of the C-N group [64]. The amide II band also shifted by 11.57 cm^−1^, which was consistent with hydrogen bond formation between WPPs and RES that increased N-H bending and C-N stretching energies [65]. In WPP-RES@CaP, calcium ion coordination further altered the N-H and C-N vibration modes, shifting the peak to 1598.34 cm^−1^ [66]. The amide III band (1300–1200 cm^−1^), associated with C-C and C-N stretching, remained centered at 1251.81 cm^−1^ but displayed increased intensity upon RES encapsulation, suggesting structural modifications caused by RES incorporation [67].

More hydrogen bonds and hydrophobic interactions were beneficial to the strength and stability of protein–polyphenol complexes [68,69]. Compared with WPP-RES, stronger non-covalent interactions between WPPs and RES were shown in WPP-RES@CaP, which indicated the formation of more stable nanoparticles [70]. Polyphenols exhibited a higher binding rate when forming covalent conjugations with proteins [71]. The introduced CaP shell strengthens the nanoparticle through enhanced non-covalent cross-linking, primarily via Ca^2+^ coordination with carboxylate groups on the peptide chains. This metal–ligand coordination acts as an additional “cross-linking” point, rigidifying the structure and thereby improving the EE of RES.

### 3.6. The Secondary Structure

**Figure 4:**
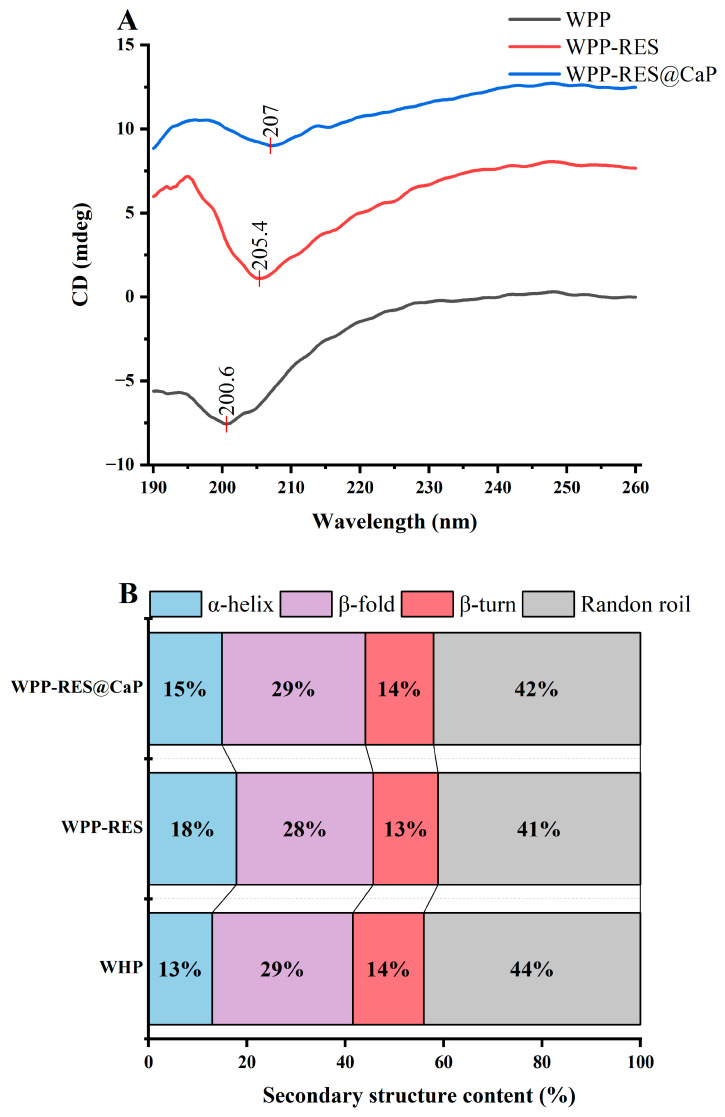
CD spectra of WPPs, WPP-RES, and WPP-RES@CaP (**A**). Secondary structure contents of WPPs, WPP-RES, and WPP-RES@CaP (**B**).

CD spectra in the far-ultraviolet region (190–260 nm) were used to analyze the secondary structures of proteins [72]. As shown in Figure 4A,B, WPPs exhibited a gradual decrease in ellipticity from 195 to 200 nm followed by an increase from 220 to 260 nm, which was consistent with a typical random coil conformation [73]. After RES incorporation, the negative peak redshifted to 205.4 nm, and the overall ellipticity increased significantly. The α-helix content increased from 13% to 18%, while β-sheet, β-turn, and random coil proportions varied within ±5%. This increase in α-helix content indicated that peptide chains reorganized into a more ordered, stable conformation upon RES loading [74]. After biomimetic mineralization, WPP-RES@CaP exhibited a further redshift of the positive and negative peaks to 195.9 nm and 207 nm, respectively, with maximal ellipticity. The α-helix content slightly decreased by 3%, while β-sheet and random coil contents increased. This rearrangement was attributed to the electrostatic interactions between Ca^2+^ ions and negatively charged carboxyl groups (Asp and Glu) [75]. The secondary structure transformation from α-helix to β-sheet would increase the contact and interaction between peptides, thereby forming a dense conformation [76]. The addition of CaP promoted this secondary structure change and then provided a denser peptide structure for embedding RES, which was more conducive to the EE of WPP-RES@CaP.

### 3.7. The Tertiary Structure

**Figure 5:**
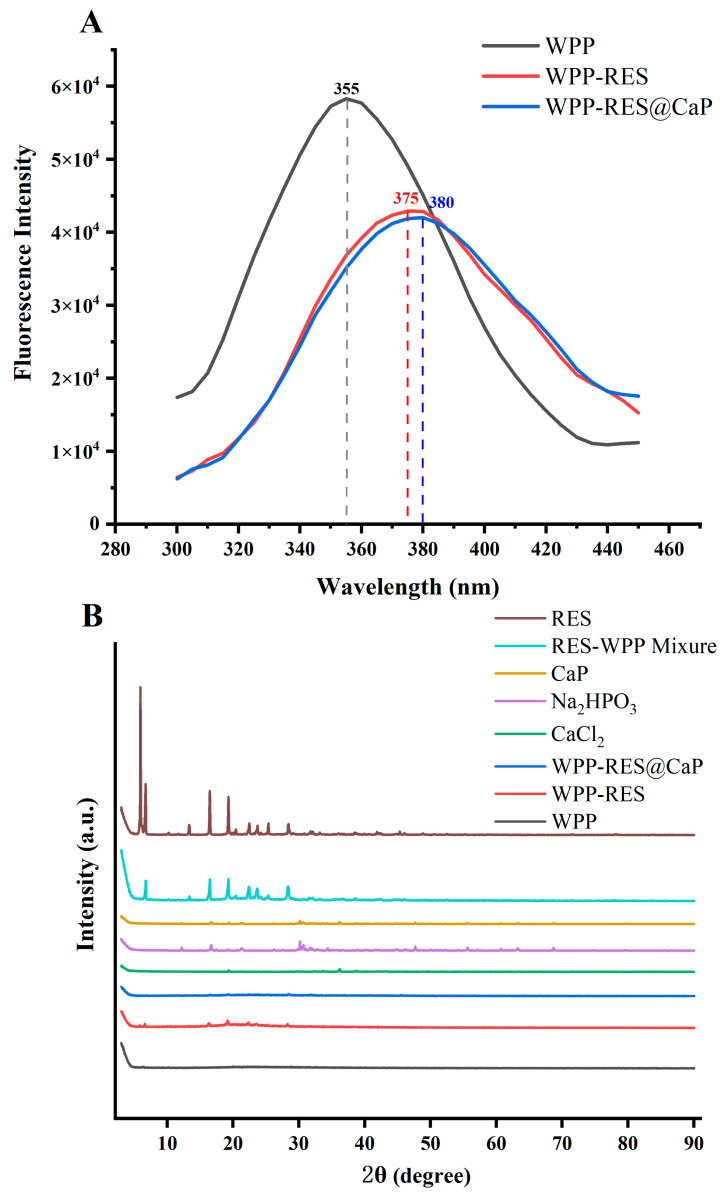
Fluorescence spectra of WPPs, WPP-RES, and WPP-RES@CaP (**A**). XRD of WPPs, WPP-RES, and WPP-RES@CaP (**B**).

Fluorescence spectroscopy was employed to evaluate conformational changes in the microenvironment of chromophoric amino acids (Tyr and Phe), thereby proving their tertiary structure [77]. As shown in Figure 5A, the fluorescence peak position and intensity of the luminescent residue are related to the hydrophobicity of the surrounding microenvironment and the conformation of the peptide chain, that is, the change in peak position reflects the polarity of the environment where the fluorophore is located, and the increase in polarity is related to the redshift [78]. The fluorescence emission peak of WPPs was observed at 355 nm, whereas that of WPP-RES redshifted to 375 nm, which was likely because RES embedded within hydrophobic regions enhanced local polarity near Trp residues [36]. The Trp residues originally buried in the hydrophobic core were exposed to a more hydrophilic microenvironment. Meanwhile, the peptide chain formed a relatively fixed folded conformation due to the embedding of RES, which not only achieved the initial encapsulation of RES but also provided a structural basis for subsequent mineralization. After mineralization, WPP-RES@CaP exhibited a further redshift to 380 nm, attributed to Ca^2+^ coordination with carboxyl and amino groups, which modulated peptide chain conformation and increased environmental polarity [58]. The further redshift of the fluorescence peak of WPP-RES@CaP compared with WPPs and WPP-RES indicated that the CaP shell modified the microenvironment of the Trp residue more significantly and enhanced the stability of the WPP-RES core. This structural adjustment helped to lock RES in the hydrophobic region of WPPs and could effectively prevent the excessive degradation of peptide chains and the direct effect on RES by the acidic environment of gastric digestion, thereby reducing the premature release of RES during the digestion process. This mechanism was consistent with the conclusion of [79], that the calcium phosphate shell stabilizes the core structure of sodium caseinate-loaded curcumin, reduces the contact of curcumin with the acidic environment, and delays the release of curcumin. Changes in fluorescence intensity can reflect the compactness of the peptide structure. The unfolding of peptide chains often exposes tryptophan residues to a more polar aqueous environment, leading to fluorescence quenching and a redshift [80]. From the perspective of fluorescence intensity changes, compared with WPPs, the fluorescence intensity of WPP-RES showed a gradually decreasing trend. The reason was that the hydrophobic interaction caused the WPP peptide chain to fold, restricting the free rotation of Trp residues and reducing the fluorescence intensity. The fluorescence intensity of WPP-RES@CaP further decreased. This might be due to the further stabilization of the steric hindrance of the CaP shell, which altered the conformation of Trp residues and reduced the exposure degree of Trp, ultimately leading to fluorescence quenching [81]. This significant fluorescence quenching essentially signified that WPP-RES@CaP had formed a more compact and rigid tertiary structure. The formation of this dense structure enhanced the physical barrier effect of the CaP shell against degradation by gastric acid and pepsin and ensured the massive release of RES in the intestinal environment [82].

### 3.8. XRD Analysis

XRD analysis was conducted to examine the crystalline characteristics of the samples [83]. As seen in Figure 5B, there were many diffraction peaks of higher intensity between 5° and 30° of RES, which indicated that it has a crystal structure [84]. Generally speaking, the carriers used to encapsulate RES need to transform their crystal form into an amorphous form to further enhance the solubility and bioavailability of RES [85]. In the physical mixture of RES and WPPs, the characteristic diffraction peaks of RES still had a relatively high intensity at the same angle, which indicated that simple physical mixing could only achieve the dispersion of RES and could not effectively encapsulate RES. In WPP-RES, the intensity of the diffraction peaks of the characteristic RES was significantly weakened, and the peak width was narrowed. This indicated that most of the RES had been successfully embedded and only small portion remained on the surface of the WPP-RES [79]. In contrast, WPP-RES@CaP exhibited only diffraction peaks corresponding to CaP precursors (Na_2_HPO_3_ and CaCl_2_) without any RES peaks, confirming the complete encapsulation of RES within the mineralized core–shell structure [86,87].

### 3.9. Molecular Docking Analysis of WPP-RES and WPP-RES@CaP

**Figure 6:**
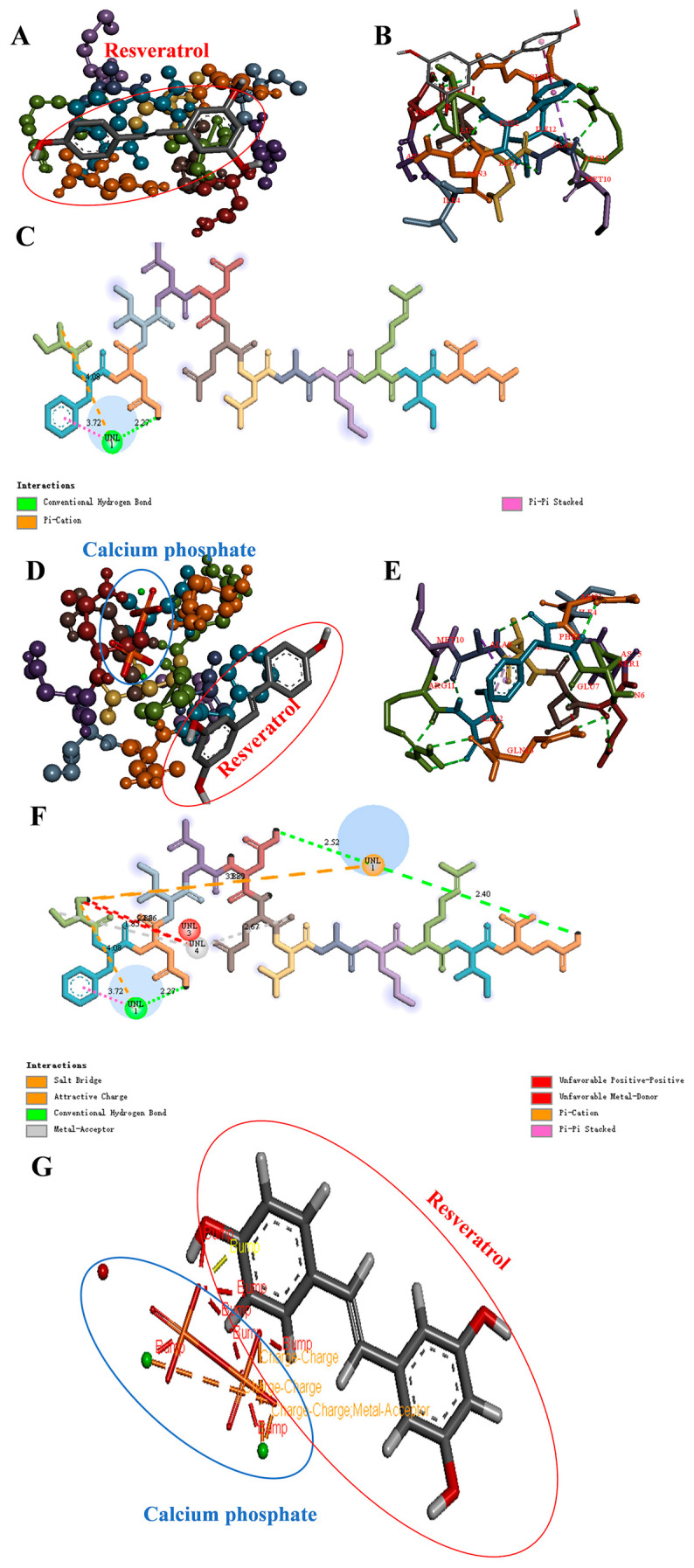
Molecular docking of WPPs, RES and CaP. The diagrams of peptide SQ (representative peptides in WPPs) and RES of the relative overall structure’s 2D interactions, and force types (**A**–**C**). The diagrams of peptide SQ, CaP, and RES of the relative overall structure’s 2D interactions, and force types (**D**–**F**). The diagrams of CaP and RES of the 2D interactions (**G**). The blue circle represents calcium phosphate, and the red circle represents resveratrol.

**Figure 7:**
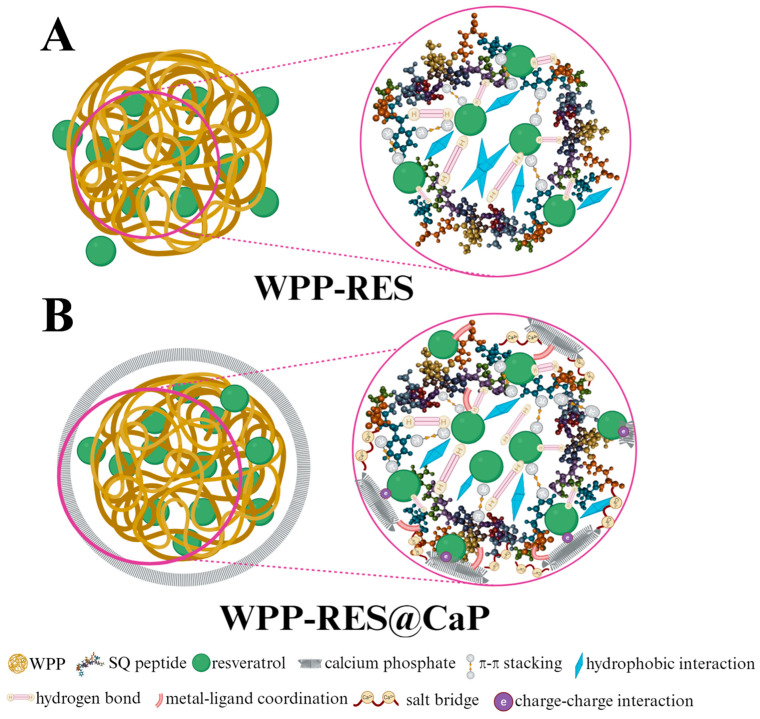
Diagram of the embedded RES mechanism in the WPP-RES binary system (**A**). Diagram of the embedded RES mechanism in the WPP-RES@CaP ternary system (**B**). The rose red circle on the right is an enlarged detail of the rose red circle marked on the left in the WPP-RES binary system in (**A**). The rose red circle on the right is an enlarged detail of the rose red circle marked on the left in the WPP-RES@CaP ternary system in (**B**).

To elucidate the reinforcement effect of CaP-mineralized WPP on encapsulated RES at the molecular level, we first selected the peptide segment SFNIDNELAMRIQ (SQ) with the highest abundance from the peptide sequences identified in the WPP (Supplementary Material S1). The binding differences before and after mineralization were analyzed through two-step molecular docking. The first step was to construct a binary system docking of peptides and RES (Figure 6A–C), and the second step was to introduce CaP to construct a ternary system docking (Figure 6D–F) to compare the changes in the embedding mechanism. The results showed that the binding affinity of SQ and RES was −4.8 kcal/mol, and the stable binding was mainly achieved through the π-π stacking interaction between Trp and Phe residues and the aromatic ring of RES, hydrogen bonds, and hydrophobic interactions. After mineralization, the ternary system exhibited multiple interactions. The binding energy of CaP and SQ was −3.8 kcal/mol, and that of SQ and RES was −4.0 kcal/mol. It should be noted that the stability of the final system of WPP-RES@CaP was not the sum of a single binary interaction, but the result of a ternary synergy effect. Although the direct interaction between SQ and RES might be weakened, the newly formed interaction between SQ-CaP and RES-CaP, as well as the resulting structural compactness, jointly contributed to a supramolecular system with the lowest global energy and greater stability. As shown in Figure 6G, the interaction between RES and CaP first included the formation of metal receptor interactions between Ca^2+^ and the oxygen-containing groups of RES. Charge–charge interactions and a space bump effect were also observed. These interactions were more conducive to WPP-RES@CaP forming a tight structure. The specific embedding mechanism diagram is shown in Figure 7A,B, and the addition of CaP altered the conformation of SQ, which made its binding energy with RES slightly lower than the direct binding energy of SQ and RES. However, the CaP was inserted into the composite cavity where SQ and RES were combined to form a more compact structure. In addition to retaining the interaction between SQ and RES, the WPP-RES@CaP embedding effect was significantly enhanced through the synergy of multiple forces such as metal–ligand coordination and salt bridges.

### 3.10. In Vitro Release Analysis of RES During Simulated Digestion

**Figure 8:**
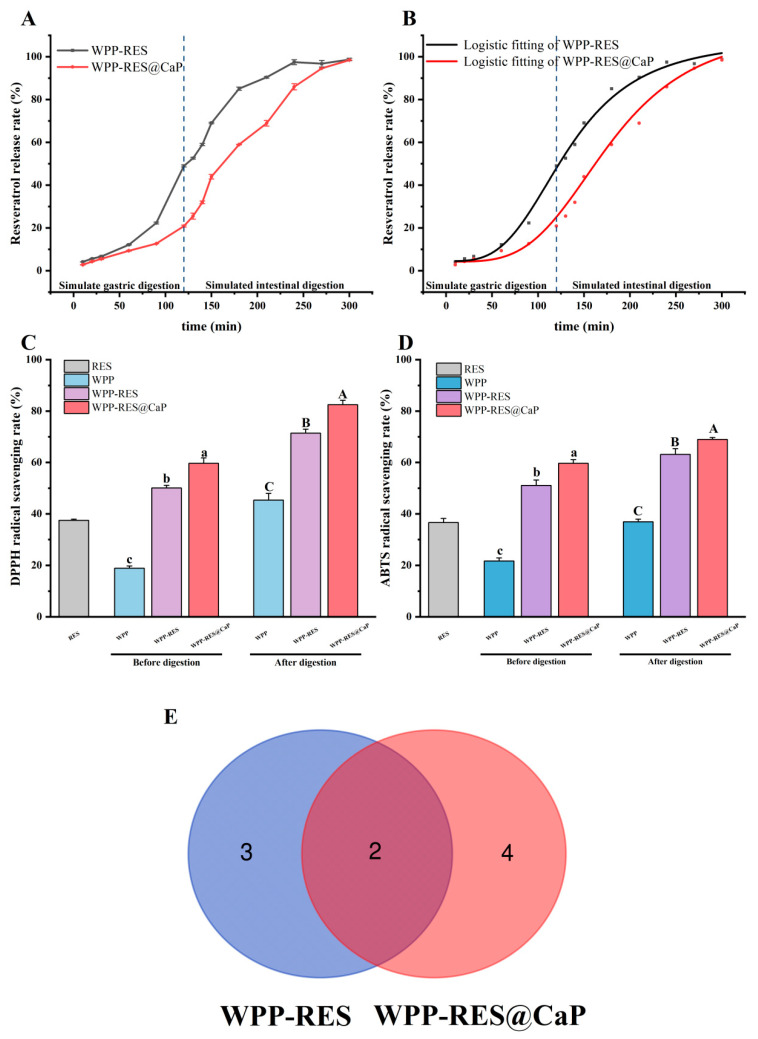
RES release efficiency of WPP-RES and WPP-RES@CaP during simulated gastric and intestinal digestion (**A**). Logistic fitting curves of RES release rate (**B**). DPPH radical-scavenging activity assay of RES, WPPs, WPP-RES, and WPP-RES@CaP before and after digestion (**C**). ABTS radical-scavenging activity assay of RES, WPPs, WPP-RES, and WPP-RES@CaP before and after digestion (**D**). Venn diagram of potential antioxidant peptides in the intestinal digests of WPP-RES and WPP-RES@CaP (**E**). The blue dashed lines in (**A**,**B**) indicate the dividing line between the gastric digestion stage and the intestinal digestion stage. The numbers in (**E**) indicate the corresponding amount of peptides in different groups. The different lowercase letters in (**C**,**D**) indicate that the DPPH and ABTS radical-scavenging activity assay components before digestion have significant differences (*p* < 0.05). The different uppercase letters in (**C**,**D**) indicate that the DPPH and ABTS radical-scavenging activity assay components before digestion have significant differences (*p* < 0.05).

Figure 8A presents the in vitro release profiles of RES from WPP-RES and WPP-RES@CaP under simulated gastrointestinal conditions. During gastric digestion, WPP-RES released RES rapidly, with 39.03% of total RES released within the first 2 h, indicating limited protection under acidic conditions. In contrast, WPP-RES@CaP exhibited less release in the gastric phase, which was attributed to the slow dissolution of the CaP shell. The CaP shell acted as a physical barrier against gastric degradation [88]. The higher zeta potential of WPP-RES@CaP suggested enhanced resistance to pepsin activity [89]. Therefore, the more negatively charged samples there are, the lower the interaction with pepsin, and the lower the release in the stomach. The higher absolute zeta potential of WPP-RES@CaP (−26.93 mV) compared to WPP-RES (−14.21 mV) suggested stronger electrostatic repulsion, which may contribute to its enhanced stability and lower RES release in the gastric phase by reducing aggregation and enzyme accessibility. During the intestinal digestion stage, the release rates of both groups significantly increased, eventually approaching 100% release. In the neutral-to-slightly alkaline intestinal environment, the CaP shell continued to dissociate [90]. At the same time, the WPP peptide chain was deeply hydrolyzed by pancreatic enzymes, and the encapsulation structure of RES was completely destroyed, which promoted the rapid release of RES.

Subsequently, we used the logistic model to fit and understand the release kinetics of RES (Figure 8B). This model could explain the release process of RES. The correlation coefficient R^2^ of WPP-RES and WPP-RES@CaP was greater than 0.99, which conformed to the S-shaped release pattern of slow release in the early stage, accelerated release in the middle stage and reaching the plateau in the later stage. EC_50_ indicated that the inflection point of the curve was the time when the release rate was maximum, and that of WPP-RES@CaP (177.5 min) was significantly greater than that of WPP-RES (132.2 min). This proved the delayed release time of WPP-RES@CaP, achieving low release of RES in the stomach and high release in the intestine, providing a guarantee for the targeted intestinal delivery of RES.

### 3.11. Antioxidant Activity

Maintaining antioxidant capacity throughout the gastrointestinal digestion process is the primary objective of delivering RES. The antioxidant activities of free RES, WPP-RES, and WPP-RES@CaP nanoparticles were evaluated before and after simulated gastrointestinal digestion using DPPH and ABTS radical-scavenging assays (Figure 8C,D). The scavenging activity assays for DPPH and ABTS radicals by free RES were 37.45% and 36.62%, respectively, while those for WPPs prior to digestion were 18.94% and 21.73%. Both RES and WPPs exhibited intrinsic antioxidant capabilities. The enzymatic hydrolysis of walnut proteins disrupts their dense spatial structure, thereby exposing active peptides that can interact with oxidants [31,91]. Prior to digestion, compared to WPPs, the scavenging activity assays of DPPH and ABTS for WPP-RES increased to 50.08% and 51.05%, respectively. This finding indicated that the encapsulation process did not diminish the inherent antioxidant activity of WPPs and RES; rather, it suggested a synergistic effect between the active groups in WPPs and RES that enhanced free radical-scavenging efficiency.

After mineralization, the scavenging activity assays for DPPH and ABTS in WPP-RES@CaP further increased to 59.68% and 59.65%, respectively. The rate of free radical-scavenging correlates with the EE of RES. EE is lower in WPP-RES than in WPP-RES@CaP due to fewer available RES molecules contributing to antioxidant activity. Secondly, after embedding and mineralization, the hydrophobic aromatic ring of RES was embedded in the hydrophobic region of the WPP peptide chain. The CaP shell stabilized the composite structure and optimized the exposure state of the antioxidant sites of RES and WPPs. The spatial arrangement of antioxidant sites such as the phenolic hydroxyl groups of RES was more conducive to the reaction with free radicals [92].

After simulated gastrointestinal digestion, the antioxidant activity of the nanoparticles was significantly enhanced. WPPs were hydrolyzed into shorter peptide fragments and free amino acids through the sequential action of enzymes such as pepsin and trypsin. These active peptides not only had an increased likelihood of interacting with free radicals due to their reduced molecular weight but also possessed relatively high redox potential, enabling them to effectively donate electrons to free radicals. This process efficiently eliminated DPPH and ABTS free radicals [93]. Zhan et al. [94] similarly observed that after the gastrointestinal digestion of Inca nut seed proteins, there was an increase in the release of small bioactive peptides, which enhanced antioxidant properties such as the ABTS radical-scavenging activity assay. Based on these findings, the further enhancement of WPP-RES and WPP-RES@CaP was attributed to the synergistic antioxidant effect resulting from RES released during digestion alongside antioxidant peptides and free amino acids.

The superior antioxidant capacity of WPP-RES@CaP after digestion is directly attributable to the protective role of the CaP shell. By shielding the core from the harsh gastric environment, the shell ensures that a larger proportion of intact RES and the WPP peptide precursors are delivered to the intestine. Subsequently, in the neutral pH of the intestine, the dissolution of the CaP shell coincides with the enzymatic hydrolysis of WPPs by pancreatin, leading to the simultaneous release of a high concentration of RES and the generation of antioxidant peptides/amino acids. This spatiotemporal co-localization and release maximize the synergistic antioxidant effect in the intestinal phase, where absorption occurs. Zhu et al. [95] encapsulated RES using corn protein and polyglycerol conjugates to enhance both chemical stability and sustained-release properties, and the antioxidant activity during simulated gastrointestinal digestion was also improved. In addition, the changes in digestion sites caused by ternary interactions can also lead to the production of different digestion products, especially active peptides and free amino acids, which will be further analyzed.

### 3.12. Peptides Analysis in Digests

Peptide identification was performed on the digested WPP-RES and WPP-RES@CaP, and 402 and 396 peptides were, respectively, identified (Supplementary Materials S1). Then, the biological activity of the peptides was predicted using the Peptide Ranker service (http://distilldeep.ucd.ie/PeptideRanker/). The URL was accessed on 22 September 2025. Peptides with a selection sort score greater than 0.50 were evaluated for subsequent activity [96]. The AnOxPePred 1.0 antioxidant peptide predict server (https://services.healthtech.dtu.dk/services/AnOxPePred-1.0/) was used to evaluate the oxidation potential of these peptides, including radical-scavenging and ion-chelating properties [97]. The URL was accessed on 22 September 2025. High-potential antioxidant peptides were screened out based on this criterion, and the number and relationship of these high-potential antioxidant peptides were demonstrated in the Venn diagram (Figure 8E). Among them, there were two high-potential antioxidant peptides shared by WPP-RES and WPP-RES@CaP, three antioxidant peptides unique to WPP-RES, and four antioxidant peptides unique to WPP-RES@CaP. The number of high-potential antioxidant peptides in WPP-RES@CaP was greater than that in WPP-RES. Studies have shown that the complexes of minerals and peptides are not easily affected by the digestive tract environment. This stability stems from the connection between the main chain structure, carboxyl groups, amino groups, and various side chains of peptides and metal ions [98,99]. The complex structure formed by CaP and peptides after mineralization enabled it to maintain the structure of peptide chain precursors in the early stage of gastrointestinal digestion and avoided premature enzymatic hydrolysis and destruction. Continuous digestion in the gastrointestinal tract accelerated the enzymatic hydrolysis process of peptide chains. It is estimated that only about 2% of peptide chains that have not formed mineral–peptide complexes could smoothly pass through gastrointestinal digestion and were converted into target bioactive peptides, while the majority would be overly hydrolyzed into inactive short fragments [100,101]. In contrast, the peptide chain of WPP-RES was completely exposed and continuously hydrolyzed during gastrointestinal digestion, thus losing the precursor basis for generating antioxidant peptides. With the protection of the CaP shell, WPP-RES@CaP might allow more polypeptide chain precursors to complete the pre-gastric digestion and undergo a large amount of enzymatic hydrolysis during intestinal digestion, and finally generate more antioxidant peptides than WPP-RES.

### 3.13. Molecular Docking of Antioxidant Peptides and DPPH/ABTS Radicals

**Figure 9:**
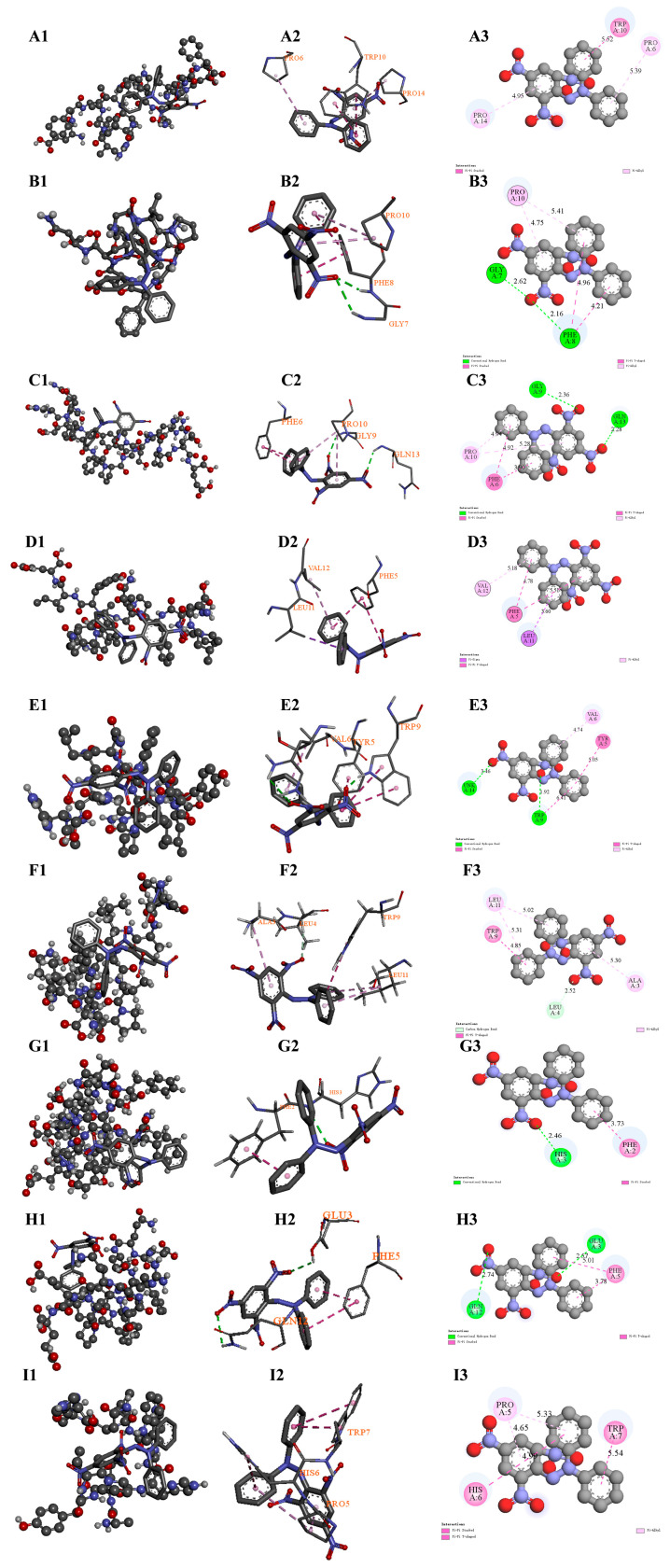
Molecular docking diagrams of potential antioxidant peptides and DPPH. Diagrams of the relative overall structure of EF and DPPH, 2D interactions, and force types (**A1**–**A3**). Diagrams of the relative overall structure of the peptide GN and DPPH, 2D interactions, and force types (**B1**–**B3**). Diagrams of the relative overall structure of the peptide QE and DPPH, 2D interactions, and force types (**C1**–**C3**). Diagrams of the relative overall structure of the peptide SE and DPPH, 2D interactions, and force types (**D1**–**D3**). Diagrams of the relative overall structure of the peptide SH and DPPH, 2D interactions, and force types (**E1**–**E3**). Diagrams of the relative overall structure of the peptide SN and DPPH, 2D interactions, and force types (**F1**–**F3**). Diagrams of the relative overall structure of the peptide AF and DPPH, 2D interactions, and force types (**G1**–**G3**). Diagrams of the relative overall structure of the peptide DE and DPPH, 2D interactions, and force types (**H1**–**H3**). Diagrams of the relative overall structure of the peptide AH and DPPH, 2D interactions, and force types (**I1**–**I3**). The dashed lines represent the intermolecular interaction forces in (**A2**–**I2**). The green dashed lines represent the hydrogen bond interaction types, the pink and purple dashed lines represent the hydrophobic interaction types, and the labeled values represent the bond lengths of the corresponding interactions in (**A3**–**I3**).

**Figure 10:**
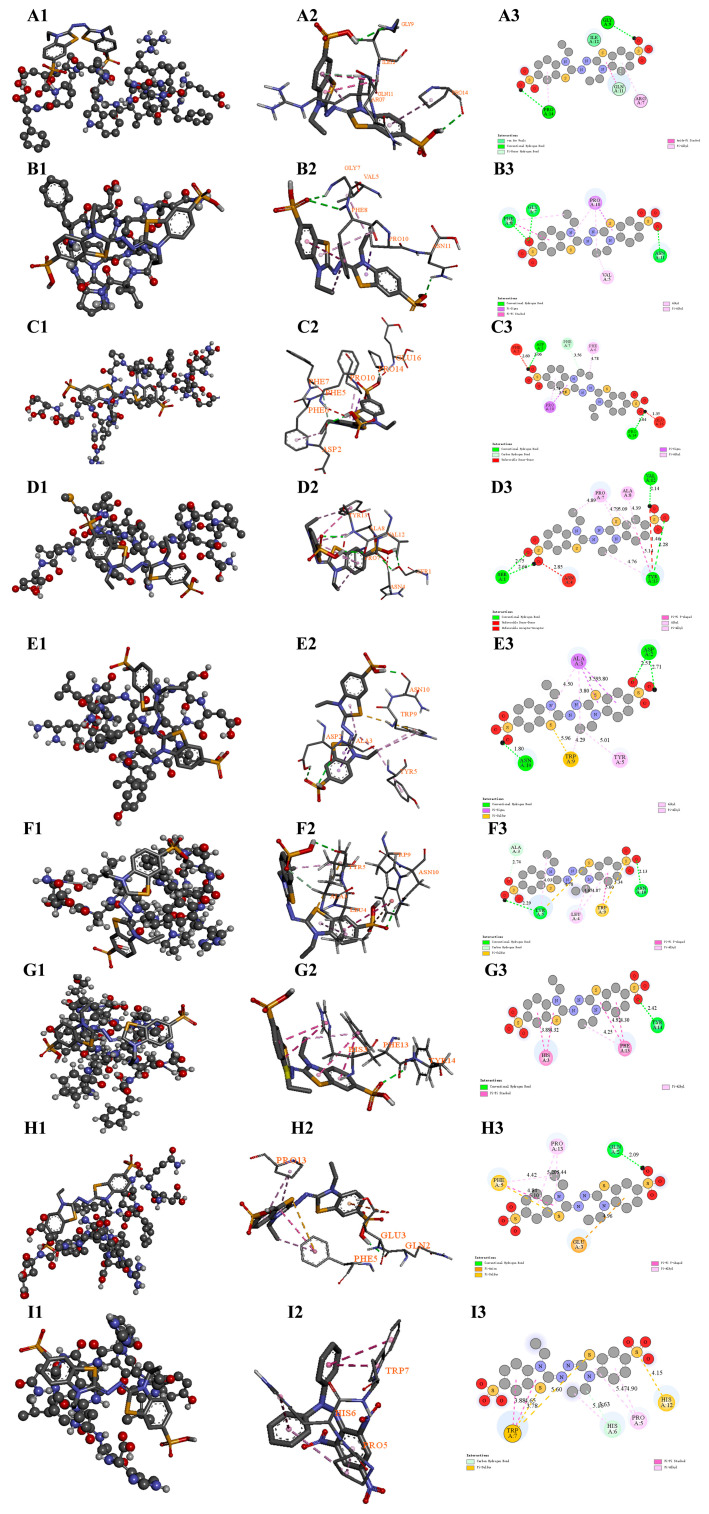
Molecular docking diagrams of peptides and ABTS. Diagrams of the relative overall structure of EF and ABTS, 2D interactions, and force types (**A1**–**A3**). Diagrams of the relative overall structure of the peptide GN and ABTS, 2D interactions, and force types (**B1**–**B3**). Diagrams of the relative overall structure of the peptide QE and ABTS, 2D interactions, and force types (**C1**–**C3**). Diagrams of the relative overall structure of the peptide SE and ABTS, 2D interactions, and force types (**D1**–**D3**). Diagrams of the relative overall structure of the peptide SH and ABTS, 2D interactions, and force types (**E1**–**E3**). Diagrams of the relative overall structure of the peptide SN and ABTS, 2D interactions, and force types (**F1**–**F3**). Diagrams of the relative overall structure of the peptide AF and ABTS, 2D interactions, and force types (**G1**–**G3**). Diagrams of the relative overall structure of the peptide DE and ABTS, 2D interactions, and force types (**H1**–**H3**). Diagrams of the relative overall structure of the peptide AH and ABTS, 2D interactions, and force types (**I1**–**I3**). The dashed lines represent the intermolecular interaction forces in (**A2**–**I2**). The green dashed lines represent hydrogen bond interaction types, the pink and purple dashed lines represent hydrophobic interaction types, the orange and yellow dashed lines represent miscellaneous interaction types and the red dashed lines represent the unfavorable interaction types in (**A3**–**I3**). The marked values represent the key lengths corresponding to the interactions in (**A3**–**I3**).

These nine high-potential antioxidant peptides in the digests of WPP-RES and WPP-RES@CaP were selected to perform molecular docking with the two model free radicals in DPPH and ABTS to verify their antioxidant activity (Figure 9 and Figure 10). The corresponding ranking scores, binding energies, and binding sites are summarized in Table 1. The peptides EFGVVPRIGWQIDPF(EF), SLPNFQPAPMLVYIE (SE), and SDALYVPHWNLNAH (SH) co-existed in both WPP-RES and WPP-RES@CaP. The peptides GPPGVPGFEPN (GN) and QEFFFPGPSRQPEE (QE) only existed in WPP-RES. The peptides SDALYVPHWNLN (SN), AFHGSGGEDPESFYRAF (AF), DQEFFFPGPSRQPEE (DE), and ALYVPHWNLNAH (AH) only existed in WPP-RES@CaP. This indicated that all these peptides have a strong potential for free radical-scavenging.

Peptides derived from WPP-RES@CaP showed significant advantages in terms of binding energy and binding sites. The binding energy of DE with DPPH and ABTS free radicals reached −5.3 and −5.5 kcal/mol, respectively. This indicated that DE could form stable bonds with both types of free radicals to eliminate them. The binding of the nine peptides to the two free radicals was mainly based on hydrogen bonds and hydrophobic interactions. However, the unique peptide QE derived from WPP-RES and the common peptide SE of both had spatially unfavorable interactions (e.g., repulsive donor–donor or acceptor–acceptor contacts) that are less conducive to stable binding. Based on the binding energy and specific interaction sites, their antioxidant mechanisms were further analyzed.

First, the interactions were primarily driven by hydrophobic forces and hydrogen bonding, consistent with a hydrogen atom transfer (HAT) mechanism [102]. The SH peptide shared by both showed strong binding affinity toward both radicals (−5.0 kcal/mol). Its interactions with DPPH and ABTS were involved the Trp9 and Tyr5 residues, and more Trp residues were also present in SN, AF, and EF from WPP-RES@CaP. According to the HAT mechanism, the phenolic hydroxyl group of tyrosine and the indole nitrogen of tryptophan acted as ideal hydrogen donors. The direct transfer of a hydrogen atom from the peptide to the radical neutralized it, thereby exerting an antioxidant effect [103].

Second, free radical-quenching was also facilitated through hydrophobic amino acid encapsulation and electron transfer (ET) mechanisms. Hydrophobic amino acids such as His, Trp, Phe, Pro, Gly, Lys, Ile, and Val exhibited high ABTS^+^ radical-scavenging ability [104], and all nine peptides contained multiple hydrophobic residues at their binding sites. These residues formed hydrophobic regions around the radicals via π–alkyl or other non-polar interactions, stabilizing the radical transition state. The binding sites of EF, GN, and AH with DPPH were particularly rich in proline residues, which contained secondary amines that effectively scavenged free radicals. Upon hydrogen abstraction, proline formed stable nitroxide radicals, thus terminating the radical chain reaction [105].

The glutamic acid in the unique DE of WPP-RES@CaP was negatively charged, and its binding sites with both free radicals involve Glu and Gln, etc. The negatively charged Glu residue could supply electrons to the ABTS radical through the electron transfer (ET) mechanism, thereby quenching the free radical, which donated electrons to further quench the radicals [106].

It was notable that the peptides released by the WPP-RES@CaP system show obvious advantages in terms of structural integrity. For instance, SN, as a truncated form of SH, maintained similar binding capabilities while having abundant binding sites and more binding interactions (carbon–hydrogen bonds). This phenomenon indicated that after mineralization forms a shell, it not only protected the key active sites of the peptide but also might optimize the sequence characteristics of the peptide, thereby enhancing the free radical-scavenging effect.

### 3.14. Analysis of Free Antioxidant Amino Acids in Digests

Besides bioactive peptides, many free amino acids were reported to have antioxidant activity. The free antioxidant amino acid compositions of WPP-RES and WPP-RES@CaP after simulated gastrointestinal digestion are compared in Table 2. The main free amino acids in the digestion products were Arg, Phe, Thr, Tyr, Leu, and Lys. This was consistent with the report by Chen et al. [107], where the proportion of arginine was the highest in the proteolytic products of walnuts. The content of free amino acids was positively correlated with antioxidant activity [108]. Compared with WPP-RES, the free antioxidant amino acid composition in WPP-RES@CaP was more abundant. The CaP shell formed by its mineralization prevented the free antioxidant amino acids from being over-hydrolyzed prematurely in the stomach. After entering the intestine, the peptides were fully enzymatically hydrolyzed, thereby generating more free amino acids. Moreover, studies had shown that Asp, Glu, Pro, Arg, His, Leu, and aromatic amino acids all possess antioxidant activity [109,110]. Among them, the relative contents of key antioxidant amino acids such as Arg, Phe, Tyr and Leu in WPP-RES@CaP were significantly higher than those in WPP-RES. Basic amino acids (Lys, Arg, and His) had been found to be hydrogen donors and possess strong free radical-scavenging activity [111]. The content of basic amino acids in WPP-RES@CaP was higher than that in WPP-RES, especially arg. Therefore, WPP-RES@CaP showed higher antioxidant activity than WPP-RES after digestion due to the greater release of antioxidant-free amino acids.

### 3.15. Mechanism Analysis

**Figure 11:**
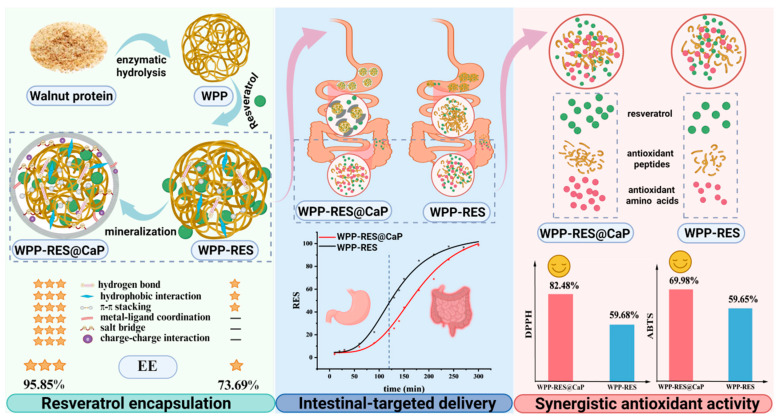
Mechanism diagram of the encapsulation, intestinal delivery, and antioxidant activity of WPP-RES and WPP-RES@CaP. In the intestinal-targeted delivery, the black dots represent the RES release efficiency of WPP-RES during digestion, and the red dots represent the RES release efficiency of WPP-RES@CaP during digestion.

Based on the above results, we systematically analyzed the encapsulation, delivery, and antioxidant mechanisms of WPP-RES@CaP at the macroscopic functional and molecular interaction levels (Figure 11). Initially, WPPs served as the matrix for self-assembled nanoparticles, which mainly encapsulate RES through π-π stacked hydrogen bonds between the aromatic rings of RES and peptides. Meanwhile, there were obvious hydrophobic interactions that embedded RES within the non-polar domains of peptides. After the introduction of CaP, the metal–ligand coordination between Ca^2+^ and peptides and the charge interaction generated by the salt bridge and phosphate anion with peptides confer a tighter structure. This structural densification effectively encapsulated RES within ternary nanoparticles, reducing RES leakage and thereby increasing the EE of RES in WPP-RES@CaP. The resulting core–shell structure of WPP-RES@CaP not only enhanced the physical barrier performance against gastric acid degradation, but also ensured to a certain extent that the structure of WPP-RES@CaP remained relatively intact during gastric digestion, which avoided premature degradation of antioxidant active components [18]. During intestinal digestion, the neutral-to-slightly alkaline environment caused the CaP shell to gradually dissociate. This weakened interactions such as salt bridges and metal coordination, disrupting the physical barrier and facilitating the release of encapsulated RES [15]. Concurrently, trypsin extensively hydrolyzed WPPs, disrupting hydrogen bonds and hydrophobic interactions between peptides and RES, thereby promoting the dissociation and release of RES from the non-polar regions of WPPs. Compared with WPP-RES, WPP-RES@CaP released RES in large quantities during intestinal digestion, while also releasing more antioxidant peptides and amino acids with antioxidant activity. Finally, the RES, antioxidant peptides, and antioxidant amino acids released after WPP-RES@CaP digestion work in synergy to counteract oxidation.

## 4. Conclusions

In this study, a nanoparticle based on biomimetic mineralized peptides (WPP-RES@CaP) was successfully constructed for the efficient encapsulation and delivery of RES. WPPs with a DH of 31.83% demonstrated the optimal EE for RES (73.69%). After biomimetic mineralization with 5 mM Ca^2+^, the EE further increased to 95.86%. The introduced CaP shell achieved a synergy between multiple forces, including hydrogen bonds, hydrophobicity, metal–ligand coordination, salt bridges and charge interactions, reinforcing the ternary structure of WPP-RES@CaP. This not only significantly enhanced EE and structural stability, but also enabled the efficient delivery of RES. In vitro digestion analysis showed that WPP-RES@CaP had a significant gastric protective effect, and the specific release of RES in the intestinal environment was high (77.57%). Its release curve conforms to the logistic model (R^2^ = 0.99), which ensured the intestinal-targeted delivery of RES and improved its bioavailability. WPP-RES@CaP released more antioxidant amino acids (such as Arg, Phe, Tyr, and Leu) and polypeptides after digestion, thereby enhancing its antioxidant activity. The free radical-scavenging activity assays of DPPH and ABTS, respectivel, reached 82.48% and 68.98%, both of which were superior to those of WPP-RES (71.43% and 63.16%, respectively). WPP-RES@CaP also released more antioxidant peptides than WPP-RES after digestion. Molecular docking studies further revealed that peptides in the digests of WPP-RES@CaP (such as peptides DE, SN, and AF) exhibited more stable binding affinities for free radicals. These peptides had optimized sequences and achieved efficient free radical-scavenging through multiple mechanisms such as hydrogen atom transfer, electron transfer, and hydrophobic encapsulation. However, there are still some limitations. The present study is mainly based on in vitro experiments. In the future, it is necessary to further verify its bioavailability and in vivo antioxidant efficacy. In addition, the stability and security of WPP-RES@CaP need to be evaluated.

## Figures and Tables

**Table 1:** Prediction of antioxidant peptides in the intestinal digests of WPP-RES and WPP-RES@CaP and their molecular docking results.

Source of Peptides	Sequence of Peptides	Ranker Score	Predicted Score for Eliminating Free Radicals	Binding Energy of DPPH (kcal/mol)	Binding Energy of ABTS (kcal/mol)	Binding Site with DPPH	Binding Site with ABTS
WPP-RES & WPP-RES@CaP	SLPNFQPAPMLVYIE(SE)	0.6512	0.5544	−4.6	−5.2	LEU11, VAL12, PHE5	TYR13, ALA8, VAL12, PRO7, ASN4, SER1
	SDALYVPHWNLNAH(SH)	0.5167	0.5703	−5.0	−5.0	VAL6, TYR5, TRP9	ASP2, ALA3, TYR5, TRP9, ASN10
WPP-RES	EFGVVPRIGWQIDPF(EF)	0.7746	0.5383	−4.6	−5.4	PRO6, TRP10, PRO14	GLY1, ILE12, GLN11, ARG7, PRO14
	GPPGVPGFEPN(GN)	0.6979	0.5343	−4.3	−4.7	PRO10, PHE18, GLY7	GLY7, VAL5, PHE8, PRO10, ASN11
	QEFFFPGPSRQPEE(QE)	0.6631	0.6329	−4.6	−5.9	PHE6, PRO10, GLY9, GLN13	ASP2, PHE5, PHE6, PHE7, PRO10, PRO14, GLU14
WPP-RES@CaP	SDALYVPHWNLN (SN)	0.7414	0.5878	−4.4	−5.1	ALA3, LEU4, TRP9, LEU11	TYR5, ALA3, LEU4, TRP9, ASN10
	AFHGSGGEDPESFYRAF (AF)	0.7042	0.5329	−4.6	−5.3	HIS3, PHE2	TYR14, HIS3, PHE13
	DQEFFFPGPSRQPEE(DE)	0.5332	0.5713	−5.3	−5.5	GLU3, PHE5, GLN12	PR013, PHE5, GLN2, GLU3
	ALYVPHWNLNAH(AH)	0.5563	0.5407	−4.6	−5.0	PRO5, TRP7, HIS6	PRO5, HIS6, TRP7, HIS12

**Table 2:** Free antioxidant amino acid contents after simulated gastrointestinal digestion in WPP-RES and WPP-RES@CaP. Different letters indicate significant differences (*p* < 0.05).

Antioxidant Amino Acids	WPP-RES (mg/20 mg)	WPP-RES@CaP (mg/20 mg)
Asp	0.069 ± 0.002 ^b^	0.099 ± 0.015 ^a^
Thr	0.628 ± 0.015 ^b^	0.800 ± 0.051 ^a^
Glu	0.368 ± 0.025 ^b^	0.460 ± 0.039 ^a^
Leu	0.789 ± 0.019 ^b^	1.070 ± 0.065 ^a^
Tyr	0.897 ± 0.028 ^b^	1.239 ± 0.109 ^a^
Phe	1.356 ± 0.03 ^b^	1.919 ± 0.171 ^a^
His	0.130 ± 0.004 ^b^	0.165 ± 0.009 ^a^
Arg	2.073 ± 0.055 ^b^	2.828 ± 0.092 ^a^

## Data Availability

The original contributions presented in this study are included in the article/Supplementary Material. Further inquiries can be directed to the corresponding authors.

## Supplementary Materials

The following supporting information can be downloaded at https://www.mdpi.com/article/10.3390/foods14244310/s1. Supplementary Material S1: Peptide sequences in the digests of WPP-RES and WPP-RES@CaP after digestion.
