# Supplementary material for: Walnut Protein Peptide Nanoparticles with Protective Mineralization: Resveratrol Encapsulation, Intestinal-Targeted Delivery and Synergistic Antioxidant Activity
Source: Foods. 2025 Dec 14;14(24):4310. doi: 10.3390/foods14244310 (PMC12732385; doi:10.3390/foods14244310)
Supplement: Supplementary file 1 [file foods-14-04310-s001.zip › foods-3996148-supplementary.pdf]

Supplementary Material S1. Peptide sequences in digests of WPP-RES and WPP-RES@CaP after digestion

WPP-RES	WPP-RES@CaP
SFNIDNELAMRIQ	RIDREEARRL
NIDNELAMRIQ	SFNIDNELAMRIQ
KAGNQGFEWVAF	NRDDQRGIIVTVEDELR
NSFNLPIL	NVDTETARRL
NRDDQRGIIVTVEDELR	KAGNQGFEWVAF
WSMTDNRVVYVL	NRDDQRGIIVT
GVAHWIYNDGESQLIVM	IVDDNGNNVFDERVKRGDVF
IVDDNGNNVFDERVKRGDVF	KTNENAMISPL
KKAGNQGFEWVAF	SESDFVSR
SVIRAMPIDVL	SVIKAMPIDVL
VEDELRVL	WSMTDNRVVYVL
KTNENAMISPL	RVEGNLQVIRPR
VFDDDLREGQLL	NSFNLPIL
RSPQSGSNMFNGFDEEFLAE	VFDDDLREGQLL
EIREGDVVAIPA	NIDNELAMR
STMRALPEDVLIN	NVDTETARRLQ
DTNFTVTSKAADDAEFGEL	RQETFLAR
AFQIPREDARRL	SESDFVSRQ
NRDDQRGIIVT	REQRGSFQNIFSGF
TARAGNNGFEYVTIK	RARNEGFEWVSF
YDTSQYNEDMKKFRKMALGS	GNLQVIRPR
RVGFQSLFSGFSEE	NRDDQRGIIVTVEDEL
VALEPSNRIEAEAGVIE	GVAHWIYNDGESQLIVM
NSFNLPILR	VFDDELREGQLL
EQRGSFQNIFSGF	NIDNELAMRIQ
SVIKAMPIDVL	TSGQPMKSPMAGY
TIPQNFGVVK	NSFNLPILRY
RALPEDVLINAY	SFNIDNELAMR
VVGENGQNVFDGEVREG	VVGENGQNVFDGEVREG
RAGNNGFEYVTIK	ENIGDPSRADIY
VVGENGQNVFDGEVRE	RVGFQSLFSGFSEE
VVGENGQNVFDGEVR	EQRGSFQNIFSGF
TSVIRAMPIDVL	VIRAMPIDVLTN
HWIYNDGESQLIVM	VVGENGQNVFDGEVRE
VFDDELREGQLL	NAQEPAQRF
WSMTDNRVVY	FSNAPRLV
QRGSFQNIFSGF	QRGSFQNIFSGF
SFNIDNELAMR	QEDDSQRGIIV
NRDDQRGIIVTVEDEL	SQRPDLQPR
NSFNLPILRY	QEDDNRRGVIV
SVIRAMPIDVLTN	RTVEPTRRWESEGGRTE

DQNPRNFYLAGNPDDEFRPQ	SVIRAMPIDVLTN
VVGENGQNVFDGEVREGQAL	VVGENGQNVFDGEVR
GIAHWIYNDGESQLIL	TSVIKAMPIDVL
AFNVDTETARRL	DQNPRNFYLAGNPDDEFRPQ
SFQMSPREAQNL	EDDSQRGIIV
RVEGNLQVIRPR	RTIEPNGLLLPQ
KAGNDGFEYVTIK	NIPVNIARRLQEDDSQRGIIV
NIPDTIAR	RGSFQNIFSGF
SESDFVSRQ	TSVIRAMPIDVLTN
GNQGFEWVAF	EYWNRNDE
NQHKLPIL	HWIYNDGESQLIVM
VIRAMPIDVLTN	VVGENGQNVFDGEVREGQA
TSVIRAMPIDVLTN	L
RTVEPTRRWESEGGRTE	SAERGVLYRN
TSVIKAMPIDVL	AYNIPDTIARRL
NVDTETARRLQ	ARAGNNGFEYVTIK
RERSPQSGSNMFNGFDEEFLAE	REQRGSFQNIFSGFSEE
RALPEDVLIN	GQNVFDGEVR
NIPDTIARRLQEDDNRRGVIV	LHDTSNQANQLDENARRFYL
ENIGDPSRADIY	A
AYNIPDTIAR	LLAEAYNIPDTIARRL
VVRRTIEPNGLLLPQ	GNPDDEFRPQ
TVEDELRVL	FSNAPRLVY
NIDNELAMR	PRDPEQRYE
RGSFQNIFSGF	RVGFQSLFSGF
RNEGFEWVSF	AFNVDTETARRL
QEDDSQRGIIVR	TARAGNNGFEYVTIK
REQRGSFQNIFSGFSEE	TFQSESSSQFR
LVYIEQGEGLMGL	AYNIPVNIA
RRLQEDDSQRGIIVR	AVMDLNNHANQLDRRFRSF
QRGSFQNIFSGFSEE	RSQQSGSNIF
KTNENAMVSPL	TIPQNFGVVKR
LLDTNNNANQLDQNPRNFYLA	SHSVIYVIR
DALEPTNRIEAEAGVIE	GIAHWIYNDGESQLIL
SDALYVPHWNLNAH	DALEPTNRIEAEAGVIE
LLAEAYNIPDTIARRL	NSLNLPILR
VALEPSNRIEAE	KSPDQSYLR
GVDLVRHTIQ	RERSPQSGSNMFNGFDEEFLA
REGDIIAFPA	E
AIRAIPEEVLANAF	RALPEDVLIN
AYNIPDTIARRL	LDMSAEKGHLFPN
TSGQPMKSPMAGY	RLQEDDNRRGVIVK
GIAHWIYNDGESQLILVE	SVIKAMPIDVLAN
EQRGSFQNIFSGFSEE	MRPDEDEQE
YSNAPQLVYIAR	TVEDELRVL

SKAGNDGFEYVTIK	TSGQPMKSPMAGYT
DLSNHANQLDRRFRSF	TSVIKAMPIDVLAN
HWIYNDGESQLIL	EDDSQRGIIVR
NDGESQLIVM	QLDQNPRNF
LDMSAEKGHLFPN	NSLNLPILRY
VFDDELRE	KTNDNAKINAL
SEELLAEAYNIPDTIARRL	AVMDLNNHAN
REQRGSFQNIFSGF	RLRENIGDPSRA
RIDREEARRL	LVYIEQGEGLMGL
TSVIKAMPIDVLAN	SAERGALYSD
SVIKAMPIDVLAN	TSGQPMKSPMAGYTSVIKAM
ALEPSNRIEAEAGVIE	PIDVL
SLSLPNFQPAPM	PSFSNAPRLVY
RGSFQNIFSGFSEE	NRDDQRGIIVTVEDELRVL
AVPAGIAHWIYNDGESQLIL	TSGQPMKSPMAGYTSVIRAM
AVPAGIAHWIY	PIDVL
TFQSESSSQFR	REGDVIAFPA
SFNIDNELAM	WVNPRVPGIL
WIYNDGESQLIVM	NELAMRIQ
FLADSFNIDNELAMRIQ	RIRHNLDTQTESDVFSR
SESDFVSR	VRQIREGDIL
VELHDTSNQANQLDENARRFY	RSPQSGSNMFNGF
LA	SHTLPVLR
IVDDNGNNVFDER	DDSQRGIIVR
IVDDNGNNVFDERVKRGDVFV	AFQIPREDARRLKFS
IPQ	LYVPHWNLN
SLSLPNFQPAPMLVY	NGQNVFDGEVR
YTPHWSMTDN	QEDDSQRGIIVR
DDDLREGQLL	DALEPTNRIEAE
ARLQVVGENGQNVFDGEVRE	TFEESQQGQSRIRPS
GQAL	DLNNHANQLDRRFRSF
GQLLTIPQNFGVVK	WSMTDNRVVY
SLPNFQPAPMLVY	LEPSNRIEAE
DTNNNANQLDQNPRNFYLA	HWIYNDGESQLIL
NIFSGFSEE	QRGSFQNIFSGFSEE
SLPNFQPAPMLVYIEQ	TSGQPMKSPMAGYTS
RSPQSGSNMFNGF	GFEYVTIK
SEELLAEAY	AGLSEYWNRNDE
GQLLTIPQNFAVVK	NDGSNPVVA
TSGQPMKSPMAGYTSVIKAMP	QLSAERGALYSDAL
IDVL	GENGQNVFDGEVR
STMRALPEDVLINAY	GQLLTIPQNFAVVK
NSLNLPILRY	SDALYVPHWNLN
SQRPDLQPR	IQLSAERGVLY
AGLSEYWNRNDE	ILYDTSNQANQL

TNSFQMSPREAQNL	NDGESQLIVM
AVMDLNNHANQLDRRFRSF	TSKAGNDGFEYVTIK
SAERGVLYRN	AFNVDTETARRLQSENDHRR
LHDTSNQANQLDENARRFYLA	SIV
YTPHWSMTDNRVVYVL	ARLQVVGENGQNVFDGEVRE
GNPDDEFRPQ	GQAL
SIPGLNPGLA	ESETSQDL
NDGSNPVVA	NGQNVFDGEVRE
NRDDQRGIIVTVEDELRVL	DERVKRGDVF
ARAGNNGFEYVTIK	SAEKGHLFPNAL
RLRENIGDPSRADIY	SDALYVPHWNLNAH
ALYTPHWSMTDNRVVYVL	TNSFQMSPREAQNL
TSGQPMKSPMAGYTSVIRAMP	RIRHNLDTQSESDFVSRQ
IDVL	RVEGNLQVIRPRWSREE
AVPAGIAHWIYNDGESQLILVE	SEELLAEAYNIPDTIARRL
QLSAERGALYSDAL	FFAVTSKAGNDGFEYVTIK
SLPNFQPAPMLVYIE	NRDDQRGRIVL
SQNLPILRWL	GQNVFDGEVRE
AFNFPAREVEKIF	GQNVFDGEVREG
SLFSGFSEE	QLSAERGALYSD
DALEPTNRIE	SAERGVLYR
KTNDNAKINALAGRLSTM	RSPQSGSNMFNGFDEE
DMSAEKGHLFPN	DTNNNANQLDQNPRNFYLA
RSPQSGSNMFNGFDEE	LDTQTESDVFSR
TFEESQQGQSRIRPS	SEELLAEAY
AGRLSTMRALPEDVLINAY	RGSFQNIFSGFSEE
RTIEPNGLLLPQYSNAPQLVYI	IVDDNGNNVFDE
A	GFEWVAFKTNDNAKIN
TSGQPMKSPMAGYTSVIK	SESDFVSRQGGRVN
PRDPEQRYE	EQRGSFQNIFSGFSEE
VFDDELREG	TDNRVVYVLR
AYNIPDTIARRLQEDDNRRGVI	REGDIIAFPA
V	NRDDQRGIIVTVE
AYNIPVNIARRLQEDDSQRGII	TVNSQNLPILRWL
V	AKGSDFNGF
TSGQPMKSPMAGYTSVIR	SDVFSRQAGRVNIV
RIDREEARRLKYN	DEYGNPIRH
ALYTPHWSMTDNRVVY	SIPGLNPGLA
TSVIKAMPIDVLANAY	DLSNHANQLDRRFR
RSQQSGSNIF	IVDDNGNNVFDERVKRGDVF
FFAVTSKAGNDGFEYVTIK	VIPQ
AFQIPREDARRLKFS	GNQGFEWVAFK
NDGESQLIL	KKAGNQGFEWVAFKTNDNA
SLPNFQPAPML	KINAL
AMYTPHWSMTDNRVVYVL	STMRALPEDVLINAY

VQIVDDNGNNVFDERVKRGD VF	LDTNNNANQLDQNPRNFYLA QMSLRDAQNL
TSGQPMKSPMAGYT	AFNFPAREVEKIF
SRREQRGSFQNIFSGFSEE	TNSFQMSPR
AGRLSTMRALPEDVLIN	TIPQNFGVVK
GQNVFDGEVR	SESDFVSRQGGRVNIVNMH
IVDDNGNNVFDE	YTPHWSMTDNRVVYVL
RLRENIGDPSRA	SAERGVLYRNA
GLLLPSFSNAPRLVY	AGNPDDEFRPQ
SEAGLSEYWNRNDE	NFPAREVEKIF
AVMDLNNHAN	YDTSNQANQL
RLQEDDNRRGVIVK	VALEPSNRIEAEAG
SWDPNNQQFQ	LHDTSNQANQL
SAERGALYSD	IDNELAMRIQ
ALAGRLSTMRALPEDVLIN	LVEDELRVL
ARLQVVGENGQNVFDGEVRE	EFQQDRHQKIRHF
DDSQRGIIVR	RDDQRGIIVTVEDELR
LEPSNRIEAE	NRDDQRGIIV
GNQGFEWVAFKTNDNAKIN	SWDPNNQQFQ
RDDQRGIIVTVEDELR	LLDTNNNANQLDQNPRNFYL A
WVAFKTNDNAKINAL	
IQLSAERGVLY	SLSLPNFQPAPMLVY
VYIEQGEGLMGL	SLPNFQPAPMLVYIEQ
DTSNQANQLDENARRFYLA	NIFSGFSEE
SVIRAMPIDVLTNSF	AERGVLYRNA
SLSLPNFQPAPML	GIAHWIYNDGESQLILVE
AVTSKAGNDGFEYVTIK	RIRHNLDTQTE
KTNDNAKINAL	PDDEFRPQ
KKAGNQGFEWVAFKTNDNAK INAL	FLADSFNIDNELAMRIQ NAPRLVYVVQ
PSFSNAPRLVYVVQ	SLPNFQPAPMLVY
NDGNEELVAF	TSVIKAMPIDVLANAY
SLNSFNLPILRY	GQNVFDGEVREGQAL
QMSLRDAQNL	GVAHWIYNDGESQ
NRDDQRGRIVL	KAGNQGFEWVAFK
YDTSNQANQL	SLPNFQPAPMLVYIE
SAIRAIPEEVLAN	RSPQSGSNMFNGFDEEFLAE
NFPAREVEKIF	DAEFLADAF
REQRGSFQNIFSGFSEELLAE	SFQMSPREAQNL
FLADSFNIDNELAMR	NDGESQLIL
DQNPRNFYLA	REGDVIAFPAGVAHW
DDQRGRIVLVEDELRVL	SFNIDNELAM
RTIEPNGLLLPQYSNAPQLVYI AR	SLNSFNLPILRY PSFSNAPRLVYVVQ
NGQNVFDGEVRE	AFHGSGGEDPESFYRAF

FSNAPRLVY	LLDTNNNANQL
TSVIRAMPIDVLTNSF	GLLLPSFSNAPRLVY
HWIYNDGESQLILVE	NVFDGEVR
KSPDQSYLR	ALEPSNRIEAE
NGQNVFDGEVR	NAQEPAQRFEAE
AGNQGFEWVAF	NERGGRITSLN
GVAHWIYNDGESQLIV	IVLVEDELRVL
DALEPTNRIEAE	SEAGLSEYWNRNDE
GQNVFDGEVRE	YTPHWSMTDN
GFEWVAFKTNDNAKIN	NVFDGEVREG
VIRAMPIDVL	SMTDNRVVYVL
QRGSFQNIFSGFSEELLAE	KTNDNAKINALA
NIINQLEREAKEL	VFDGEVREGQAL
YTPHWSMTDNRVVY	TFQSESSSQFRGE
EQRGSFQNIFSGFSEELLAE	QGEGLMGL
NDGSNPVVAI	TVNSQNLPILR
WVSFKTNENAMVSPL	SAEKGHLFPN
NVFSGFDAEFL	SVIRAMPIDVLTNSF
GVAHWIYNDGESQ	SLSLPNFQPAPM
VVRRTIEPNGLLLPQYSNAPQL	SSESFGWQR
VY	SLFSGFSEE
ILYDTSNQANQL	NDGSNPVVAI
SFNMPREEIEEIF	SNQANQLDENARRFYLA
SDDNGIVYPW	IQNRDDQRGIIVTVEDELR
SEELLAEAYNIPVNIARRL	AYNIPDTIAR
TFQSESSSQFRGE	HDTSNQANQLDENARRF
GFEWVSFKTNE	VIRGNARLQVVGENGQNVFD
DNAKINALAGRLSTM	GEVRE
LFSGFSEE	AYNIPVNIARRLQEDDSQRGII
TDNRVVYVLR	V
TSGQPMKSPMAGYTS	LFSGFSEE
SLLDTNNNANQLDQNPRNFYL	ENIGDPSRA
A	HNLDTQTESDVF
IVDDNGDNVFDERVKRGDVY	SEAGLSEYWNR
VIRGNARLQVVGENGQNVFDG	SREFQQDRHQKIRHF
EVRE	TESDVFSR
GFEWVAFKTN	LDTNNNANQLDQNPRNF
VALEPSNRIEAEAG	DAQNLKNSRGHQSF
QLNRLDALEPTNRIEAEAGVIE	WVNPRVPGILE
RIRHNLDTQSESDFVSRQ	VELHDTSNQANQLDENARRF
LHDTSNQANQL	YLA
NAQEPAQRFEAE	AVTSKAGNDGFEYVTIK
LDTQTESDVFSR	GNQGFEWVAFKTNDNAKINA
SERPSYSNQFGQFF	L
GFEWVSFKTNENAMVSPL	LLDTNNNANQLDQNPRNF

KAGNQGFEWVAFK	AYQMSLRDAQNL
GFEWVSFK	REQRGSFQNIFSGFSEELLAE
GNARLQVVGENGQNVFDGEV	RIDREEARRLKYN
REG	NRDDQRGIIVTV
LSFNMPREEIEEIFE	YNDGSNPVVA
AGYTSVIRAMPIDVL	HNLDTQTESDVFSR
GQNVFDGEVREG	NGQNVFDGEVREGQAL
NGQNVFDGEVREGQAL	FLAGGEPRQE
LLAEAYNIPDTIAR	RIQNRDDQRGIIVTVEDELR
SLNSFNLPIL	NDNAKINALAGRL
RTIEPNGLLLPQYSNAPQLVY	SLPNFQPAPML
RVGFQSLFSGFSEELLAE	EYWNRNDEQFR
REGDIIAFPAGVAHW	GAKSPDQSYLR
GFEWVAFKTNDNAKINAL	AMYTPHWSMTDNRVVY
VAHWIYNDGESQLIVM	SRDQHQKVREIREGDVVAIPA
IDNELAMRIQ	IEQGEGLMGL
DDQNPRDPEQRYE	DLSNHANQL
RGSFQNIFSGFSEELLAE	AFNFPAREVEKIFRNQDQE
AERGVLYRNA	TNDNAKTNPL
NDNAKINALAGRLSTM	AGSIPGLNPGL
DAQNLKNSRGHQSFLLS	FSRQETFLAR
SHTLPVLR	GVAHWIYNDGESQLIV
RALPEDVLINAYRIDREEARRL	KSPMAGYT
DMSAEKGHLFPNAL	GENGQNVFDGEVRE
IVLVEDELRVL	GENGQNVFDGEVREGQAL
TSGQPMKSPM	ESELLMRVKEQ
VFDGEVREGQAL	VFDDDLRE
EFGVVPRIGWQIDPF	WIYNDGESQLIVM
DSDDNGIVYPW	RTIEPNGLLLPQYSNAPQLVYI
LSFNMPREEIEEIFESQ	AR
SNTPDDSYDPL	EPSNRIEAEAGVIE
HNLDTQTESDVF	QLSAERGALY
LDMSAEKGHLFPNAL	AVMDLNNHANQL
TVPQNFAVIK	NDNAKINALAGRLSTM
TVNSQNLPIL	TSVIRAMPIDVLTNSF
RARNEGFEWVSFKTN	TEQAGRISTVNSQ
WLEEEDDTMPPGDAPQTA	LLAEAYNIPDTIAR
NTPDDSYDPL	AYRIDREEARRL
SFNMPREEIEEIFESQ	WVSFKTNENAMVSPL
DAQNLKNSRGHQSF	HGSGGEDPESFYRAF
EFQQDRHQKIRHF	DLNNHANQLDRRFR
ALYTPHWSMTDN	NQGFEWVAF
SGFDADFLAD	NDGNEELVAF
AHWIYNDGESQLIVM	DDSQRGIIV
FDSDDNGIVYPW	DQNPRNFYLA

QSENDHRRSIVRVE	REGDVIAFPAGVAH
TIPQNFGVVKRA	IRPDEDRQE
RIQNRDDQRGIIVTVEDELR	EYGNPIRH
WVNPRVPGILE	TSGQPMKSPMAGYTSVIK
TEQAGRISTVNSQ	GGEDPESFYRAF
TMRALPEDVLIN	ALYVPHWNLNAH
SGPPNLIGPY	EIREGDVVAIPA
SGFDADFLADAF	HWIYNDGESQ
WLQLSAERGALYSDAL	VYIEQGEGLMGL
NDNAKINALAGRL	NVDTETARRLQSN
GLLLPQYSNAPQLVYIAR	NLPILRYIQLSAE
SNQANQLDENARRFYLA	NSFNLPILR
RALPEDVLINAYRID	GSIPGLNPGLA
IYNDGESQLIL	HWIYNDGESQLILVE
AVIKKAGNQGFEWVAF	NAQEPAQRFEAEAGLTE
KTNDNAKINALA	SFNIDNELAMRI
GAKSPDQSYLR	GAKSPDQSYL
DQEFFFPGPSRQPEE	GFEWVAFKTNDNAKINAL
SLFSGFSEELLAE	YNDGSNPVVAI
NAQEPAQRFEAEAGLTE	SFQRDRHQKIRHF
NSFNLPILRYIQ	VVGENGQNVFDGEVREGQ
QVVGENGQNVFDGEVRE	WVAFKTNDNAKIN
SLPNFQPAPM	GFEWVSFK
TVNSQNLPILR	ALAGRLSTMRALPEDVLIN
LDTQSESDFVSRQGGRVNIV	GNARLQVVGENGQNVFDGE
NLPILRWL	VRE
NDGESQLILVELH	LDTQSESDFVSRQGGRVNIV
AVEGINKIVTGDLISL	AVPAGIAHWIYNDGESQLILV
IVDDNGDNVFDER	E
VVGENGQNVFDG	DMSAEKGHLFPNAM
SFNIDNELAMRI	NTPDDSYDPL
AMPIDVLANAYQMSLR	GENGQNVFDGEVREG
HWIYNDGESQLIV	LDTQTESDVF
GAKSPDQSYL	TVNSQNLPILRW
AFQIPREDARRLK	DTNNNANQLDQNPRNF
GYTSVIRAMPIDVL	SLNSFNLPIL
NIFSGFSEELLAE	DDQNPRDPEQRYE
GGEDPESFYRAF	SNTPDDSYDPL
WIYNDGESQLIL	VIPQFFAVTSKAGNDGFEYVT
ERSPQSGSNMFNGFDEEFLAE	IK
SGFDAEFLADAF	YLDMSAEKGHLFPN
DAQNLKNSRGHQSFLL	EYHSPADLILPF
GQNNIINQLEREAKEL	QRGSFQNIFSGFSEELLAE
FAVTSKAGNDGFEYVTIK	AYNIPDTIARR
TVNSHTLPVLRWL	GIAHWIYNDGESQ

SERPSYSNQFGQFFE	AGNQGFEWVAF
GIAHWIYNDGESQ	NVFSGFDAEFL
AMPIDVLTNSFQMSPREAQNL	VVGENGQNVFDGE
FLADSFNIDNE	KAGNQGFEWVAFKT
LDTQSESDFVSRQ	DLNNHANQL
NGLVPIVEPE	SGPPNLIGPY
RDETTLFSSESFGW	NIINQLEREAKEL
NNIINQLEREAKEL	LWEESEDEFQ
NDGESQLILVEL	LDTQSESDFVSR
LPQGLAALLSKVE	SKAGNDGFEY
YGTFLPVGDSE	EQRGSFQNIFSGFSEELLAE
HFREGDVIAFPAGVAH	AVMDLNNH
MFNGFDEEFLAE	GSFQNIFSGF
RSPQSGSNMFNGFDEEFL	WVAFKTNDNAKINAL
ALYTPHWSM	AIRAIPEEVLAN
FKTNENAMISPL	IYNDGESQLIVM
RSPQSGSNMFNGFDEEFLA	LDTQTESDVFSRQ
ETFLARSR	YLAGNPHQQ
TNDNAKTNPL	RVGFQSLFSGFSEELLAE
NYNQALLQLEHAL	DQEFFFPGPSRQPEE
PQNFGVVK	GYTSVIRAMPIDVL
FSNAPRLVYVVQ	LWEESEDE
NNGFEYVTIK	AVEGINKIVTGDLISL
QLLTIPQNFAVVK	WLQLSAERGALYSDAL
AKSPDQSYL	IEQGEGLM
SREFQQDRHQKIRHF	YSNAPQLVYIAR
NSLNLPILRYIQ	GFEWVSFKTNENAMVSPL
SLLDTNNNANQLDQNPRNF	SGFDADFLAD
TVNSQNLPILRWLQ	NLPILRWL
PNSFSNLTQLIM	EDEDFETR
SKAGNDGFEY	SGFDADFLADAF
LQVVGENGQNVFDGEVREG	SGFDAEFLADAF
LHDTSNQANQLDEN	RGSFQNIFSGFSEELLAE
IEQGEGLM	AIRQTVQPN
LQVVGENGQNVFDGEVR	AIRQTVQPNSL
LQVVGENGQNVFDGEVRE	AISDYIIFQVESGAH
RSPQSGSNMFNGFDEEFLAESF	QVVGENGQNVFDGEVR
HFREGDVIAFPA	RAGNNGFEYVTIK
QDQEFFFPGPSRQPEE	KKAGNQGFEWVAF
NLPILRYIQL	RLRENIGDPSRADIY
GPPGVPGFEPN	IGIGTPPQNF
PSFSNAPTLIY	IVDDNGNNVFDER
TYESETSQDL	QVVGENGQNVFDGEVRE
DLSNHANQLD	NDGESQLILVELH
EDEDFETR	NVAKSLCWKKLIEKDD

RVGFQSLFSGF	GFEWVAFKTN
NEGFEWVSFK	PQNFGVVK
NGQNVFDGEVREG	NYNQALLQLEHAL
ISLLDTNNNANQLDQNPRNFYLA	WLQLSAERGALY
TVNSQNLPILRW	VVRRTIEPNGLLLPQYSNAPQLVY
AISDYIIFQVESGAH	RLRENIGDPSRADIYTEQ
QSQRPDLQPR	HWIYNDGESQLIV
WLEEIEMPENSEIDEIEYL	LQVVGENGQNVFDGEVRE
AERGVLYRNALVAPL	SVMRALPEDVLINAY
WVNPRVPGIL	TARAGNNGFEYVTIKTS
RARNEGFEWVSF	TESDVFSRQ
TLQQLGMMQQHAW	
QIPREDARRLK	
SVMRALPEDVLINAY	
KSGEFVELSGGLHGGSEPPAFQ	
LDFLESQLQSGFSEE	
